# Evaluating the Effectiveness of Internet-Based Communication for Public Health: Systematic Review

**DOI:** 10.2196/38541

**Published:** 2022-09-13

**Authors:** Elisabetta Ceretti, Loredana Covolo, Francesca Cappellini, Alberto Nanni, Sara Sorosina, Andrea Beatini, Mirella Taranto, Arianna Gasparini, Paola De Castro, Silvio Brusaferro, Umberto Gelatti

**Affiliations:** 1 Section of Public Health and Human Sciences, Department of Medical and Surgical Specialties, Radiological Sciences and Public Health, University of Brescia Brescia Italy; 2 Post-graduate School of Public Health, University of Brescia Brescia Italy; 3 Italian National Institute of Health Rome Italy

**Keywords:** internet-based communication, websites, social media, public health, efficacy, systematic review, communication, internet-based, health information, exchange, health care, web-based, campaigns

## Abstract

**Background:**

Communicating strategically is a key issue for health organizations. Over the past decade, health care communication via social media and websites has generated a great deal of studies examining different realities of communication strategies. However, when it comes to systematic reviews, there is fragmentary evidence on this type of communication.

**Objective:**

The aim of this systematic review was to summarize the evidence on web institutional health communication for public health authorities to evaluate possible aim-specific key points based on these existing studies.

**Methods:**

Guided by the PRISMA (Preferred Reporting Items for Systematic Reviews and Meta-Analyses) statement, we conducted a comprehensive review across 2 electronic databases (PubMed and Web of Science) from January 1, 2011, to October 7, 2021, searching for studies investigating institutional health communication. In total, 2 independent researchers (AN and SS) reviewed the articles for inclusion, and the assessment of methodological quality was based on the Kmet appraisal checklist.

**Results:**

A total of 78 articles were selected. Most studies (35/78, 45%) targeted health promotion and disease prevention, followed by crisis communication (24/78, 31%), general health (13/78, 17%), and misinformation correction and health promotion (6/78, 8%). Engagement and message framing were the most analyzed aspects. Few studies (14/78, 18%) focused on campaign effectiveness. Only 23% (18/78) of the studies had an experimental design. The Kmet evaluation was used to distinguish studies presenting a solid structure from lacking studies. In particular, considering the 0.75-point threshold, 36% (28/78) of the studies were excluded. Studies above this threshold were used to identify a series of aim-specific and medium-specific suggestions as the communication strategies used differed greatly.

**Conclusions:**

Overall, the findings suggest that no single strategy works best in the case of web-based health care communication. The extreme variability of outcomes and the lack of a unitary measure for assessing the end points of a specific campaign or study lead us to reconsider the tools we use to evaluate the efficacy of web-based health communication.

## Introduction

### Background

Communicating strategically requires a clearly defined strategy with specific goals established in advance. The core agenda of strategic communication is the analysis and explanation of intentional and purposeful communicative relationships between organizations and the public [1]. That being said, it is important to point out that organizations make strategic decisions about the level and nature of resources they devote to such efforts, modulating their tone and tenor of communication depending on the audience they want to appeal to [2]. In the field of institutional health care communication, this theme is especially relevant, as illustrated by the *WHO fact sheet on the Strategic Communications Framework for Effective Communications* [3]. In this document, the World Health Organization wanted to establish a framework to describe a strategic approach for effectively communicating health care–related information, advice, and guidance across a broad range of health issues. This resulted in the identification of 6 key principles: accessibility, actionability, credibility and trustworthiness, relevancy, timeliness, and understandability. However, we can observe that these guidelines are not specific enough and, on the contrary, appear to be too broad. Looking at the existing literature, it is also possible to observe a lack of specific evidence regarding the effectiveness of those studies on institutional health care communication. Therefore, it is important to be able to effectively communicate with the public at large. This would allow public health officials to minimize damage and possibly prevent widespread illness and diseases. Providing accurate and verifiable information is also paramount to keep the public informed and allow them to take the appropriate action. One of the main aims of this systematic review was to analyze a corpus of studies on institutional health care communication to see whether it is possible to extrapolate aim-specific key points based on these existing studies.

As of January 2021, there were 4.66 billion active internet users on the web; 59.5% of the entire population [4]. With the dramatic increase in internet access, there has been a parallel increase in the use of the internet as a platform for the delivery of public health interventions across a wide range of conditions and population segments [5]. Over the past decade, health care communication via social media and websites has generated a great deal of studies examining different realities of communication strategies [6-8]. However, this vast diffusion of internet health care communication is a double-edged sword, as demonstrated by the *infodemic* [9] occurring during the ongoing COVID-19 pandemic. In this context, along with the diffusion of trustworthy information and guidelines from governments and health care organizations, a massive wave of false information has also spread. Although misinformation has spread throughout history, social media and technological advances in communication have amplified its impact, making it difficult for information from official sources to spread effectively without being drowned by this false information [10]. Thus, the absence of specific guidelines to effectively communicate via social media or websites has posed a problem that is yet to be addressed properly, as public health institutions have struggled to find their footing in this area, as well as a unified communication strategy for the diffusion of official messages [11].

The current evidence on internet-based health care communication appears to be rather fragmentary and localized according to topic- and platform-specific criteria. A number of other systematic reviews were published over the past 10 years [12-18]. In particular, the systematic review by Moorhead et al [18] claims that there is a lack of communication about the uses, beliefs, and limitations of social media for health communication. In total, 2 other systematic reviews [14,17] deal with providing evidence of effectiveness for studies on web-based communication, concluding that effectiveness was only sparsely reported and reach was only being assessed among those involved in the research process. Going into even more specific accounts as related to web-based health communication, the studies by Alamoodi et al [12], Kim [15], and Lehto and Oinas-Kukkonen [16] deal with the public’s perception of this type of communication in 3 different instances: trust in websites, persuasive features of web-based interventions, and application of sentiment analysis. Even in this case, the conclusions leave a substantial gap to be filled with future research. One last study [13] focuses on a completely different aspect of social media communication by basing its text collection on studies on specific social media platforms rather than on specific interventions made on the web at large. More specifically, picture-based social media such as Instagram, Pinterest, Tumblr, and Flickr are the platforms taken into account. In this case, the focus is on images used as vehicles for health care communication. However, most of these studies appear to be observational, and only few provide more specific intervention tools.

### Objectives

The objective of this systematic review was to form a more comprehensive and extensive account on the matter of web-based health communication (especially making reference to national health care institutions and nongovernmental organizations) than the aforementioned studies through a comprehensive bibliographic search of articles dealing with this topic over multiple platforms. In addition to identifying the most relevant articles on this matter, this review tried to define a series of key points as comprehensively as possible that can be applied to health campaigns spread through websites or different social media by health organizations.

## Methods

This systematic review was carried out according to the PRISMA (Preferred Reporting Items for Systematic Reviews and Meta-Analyses) guidelines [19].

### Information Sources

The literature search covered the period from January 1, 2011, to October 7, 2021—as web-based communication has undergone a rapid and drastic change over the past decade and research published before this date can appear to be rather obsolete and misleading for the scope of this study—and was carried out using electronic databases. The research process was separated into 2 parts: (1) research via electronic databases (PubMed and Web of Science) and (2) research through analysis of relevant systematic reviews (bibliographies were analyzed, and suitable articles were assessed for eligibility).

### Search Strategies and Study Selection

A bibliographic search was conducted on PubMed and Web of Science using the following search string: (*Social Media* OR *Twitter* OR *Facebook* OR *Instagram* OR *Website*) AND (communication strategy*) AND (*health* OR *public health* OR organization* OR agenc* OR *risk*) NOT (hospital* OR practitioner*).

Duplicates were identified via Zotero (Corporation for Digital Scholarship) [20,21] and eliminated.

Search results were initially evaluated based on the title and abstract by 2 independent reviewers (AN and SS), which resulted in the exclusion of all clearly irrelevant articles. In case of disagreement between the 2 parties, a third member of the team (FC) was included to resolve all conflicts.

All studies identified in this preliminary evaluation phase were considered eligible for assessment based on the exclusion and inclusion criteria stated in the following section.

### Inclusion and Exclusion Criteria

We included articles according to the following criteria: peer-reviewed or book section; published between January 1, 2011 and October 7, 2021; and written in English.

As for the research topic, we included research papers focused on social media– or website-based institutional communication strategies for health care promotion and health care promotion campaigns organized by public authorities or health care–related nongovernmental organizations spread via social media or websites and that illustrated their communication strategies.

We excluded all publications related to communication strategies applied to physician-patient communication, telemedicine, and hospital portals addressing patients; articles related to marketing communication and private institutions were also left out. The exclusion criteria also comprised qualitative studies and preliminary and exploratory articles.

### Quality Assessment

The methodological quality of each study was assessed by 2 of the authors (FC and AB) using the Kmet tool for evaluating quantitative and qualitative research [22]. A score between 0 and 1 was assigned to each paper based on a series of questions related to the type of study. Examples of items include the following: description of the research objective, appropriateness of the study design, description of participant characteristics, blinding, sample size, analytic methods, estimates of variance, control of confounding factors, and reporting of results and conclusions. A score of >0.75% was considered good quality, 0.55% to 0.75% was considered adequate quality, and <0.55% was considered poor quality. Any disagreements were resolved through discussion among the authors until a consensus was reached. Interrater reliability for the Kmet ratings was established based on κ calculations.

To further analyze the difference in the distribution of studies according to their quality, chi-square or Fischer exact tests were carried out analyzing the differences between the number of studies above and below the 0.75-point threshold.

### Data Collection and Analysis

We categorized the studies into 4 groups according to the topic addressed: crisis communication, health promotion and disease prevention, general health, and misinformation correction and health literacy. For public health emergencies, risk communication includes a range of communication capacities with the aim of encouraging positive decision-making, positive behavior change, and the maintenance of trust. This definition seems to be applicable to both the crisis communication and health promotion and disease prevention categories [23]. However, there is an important difference in the aims of these 2 types of communication: in the case of health promotion and disease prevention, health messaging advocates for an ongoing behavior change (ie, a behavior that requires an individual to keep up with a habitual activity); differently, in the case of crisis communication, the behavior change that is promoted is episodic and valid only in the case of a specific emergency [24]. Finally, those studies not dealing with any of the aforementioned categories were classified under general health. This was the case for studies analyzing the impact of a certain communication theory on communication or studies that globally analyzed a certain communication medium.

We further categorized articles according to their primary evaluation aspects. These are *engagement, message framing,* and *campaign effectiveness*. First, *engagement* is defined as a psychological and behavioral attribute of connection, interaction, participation, and involvement designed to elicit an outcome at the individual or social level [25]. In particular, in the case of social media, it is closely related to the concept of interaction with posts, where engagement is measured as the sum of the number of likes, comments, and shares [26]. Second, *campaign effectiveness* is closely related to the change in one’s attitudes and behaviors regarding a certain issue [27]. Finally, *message framing* constitutes the way in which a certain message is expressed and carried out (eg, gain- or loss-framed messages), and its content and connotative structure can prove effective in motivating individuals to engage in health-related behaviors [28].

Regarding study design, we categorized as *experimental* those studies where a specific intervention was recorded. More specifically, this can mean subjecting a group of individuals to different iterations of a post to see how its framing affects them. In the *observational* category, we included cross-sectional studies aimed at analyzing how a population sample reacted to a specific intervention (eg, the implementation of a certain campaign). Finally, *content analysis* refers to the analysis of a specific collection of posts with regard to their characteristics and the engagement generated.

To further analyze the effects of the threshold applied to the studies in this systematic review, the Fischer exact test was carried out analyzing the differences between the number of studies above and below the 0.75-point cutoff for all communication media.

**Figure 1:**
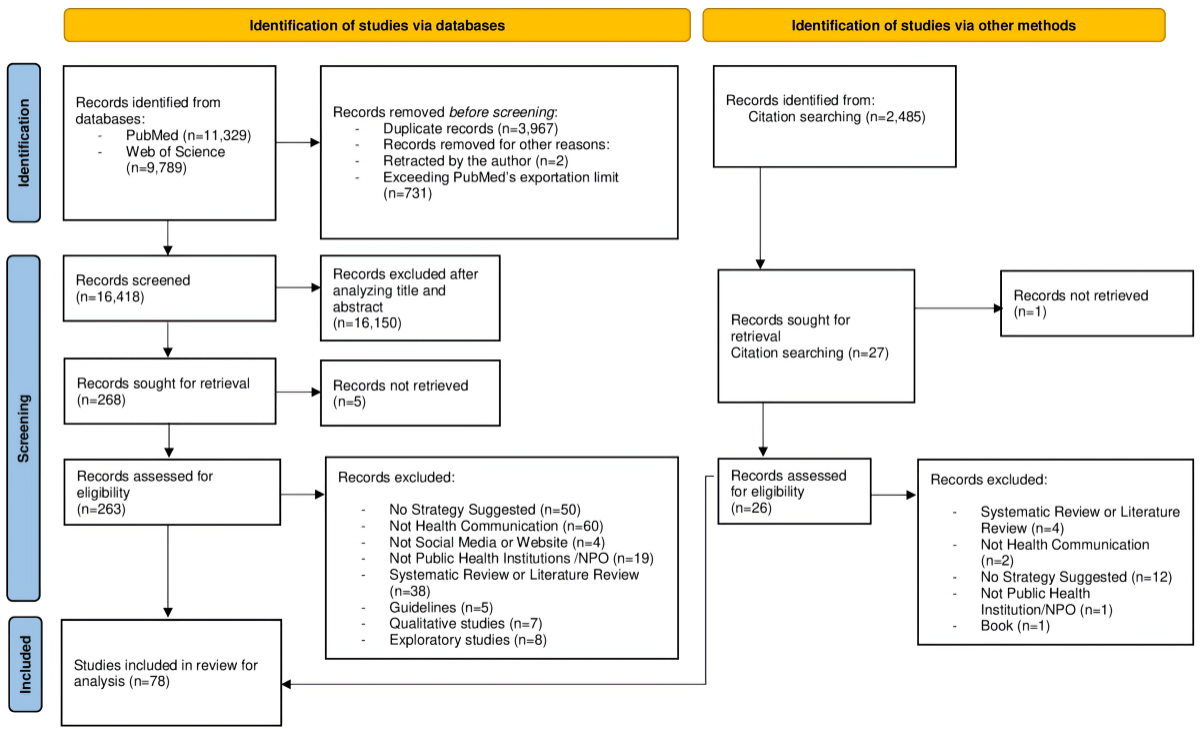
PRISMA (Preferred Reporting Items for Systematic Reviews and Meta-Analyses) flowchart for this systematic review.

Reported in Figure 1 is the PRISMA flow diagram for this specific systematic review.

## Results

### Overview

Of the 288 relevant articles selected, 78 (27.1%) met the inclusion criteria and were considered for this systematic review. These articles were divided into 4 categories according to the primary evaluated aspect of the study: (1) studies on crisis communication, (2) studies on health promotion and disease prevention, (3) studies on general health, and (4) studies on health literacy and misinformation correction. In particular, the latter category comprised studies on topics that appeared only a few times throughout the corpus, making it difficult to group them by themselves. Overall, the Kmet score of the evidence reviewed ranged from 0.40 to 0.93, with an average score of 0.75 (SD 0.10) and a correlation coefficient of 0.80 between the 2 reviewers.

### Studies on Crisis Communication

#### Overview

We selected 24 studies dealing with health care institution interventions on crisis communication (Table 1), of which 12 (50%) were carried out in the United States, 3 (13%) were carried out in China, and 3 (13%) were carried out in Canada. Engagement was the most represented primary evaluated aspect (17/24, 71%), followed by message framing (4/24, 17%) and campaign effectiveness (3/24, 13%). In this category, the Kmet evaluation score resulted in an average of 0.73 points, with a correlation coefficient of 0.85.

Overall, in this group, studies focused for the most part on the analysis of collections of posts and inquiries (21/24, 88%), whereas the rest (3/24, 13%) focused on the analysis of people as participants. Consequently, the design of these studies included a high percentage of content analyses (14/24, 58%), observational studies (5/24, 21%), and network analyses (4/24, 17%). As for the media channels analyzed in this group, 83% (20/24) of the studies focused on only 1 communication medium, whereas 17% (4/24) dealt with multiple media. Finally, half of the studies (12/24, 50%) referred to a specific communication theory.

**Table 1:** List of studies on crisis communication (see Multimedia Appendix 1 [24-101] for more details; N=24).

Primary evaluated aspect and communication medium		Reference	Studies, n (%)
**Engagement**			
	Facebook	Dimanlig-Cruz et al [31] Lwin et al [37]	2 (8)
	Twitter	Kim et al [25] Dimanlig-Cruz et al [31] Hagen et al [32] Lauran et al [33] Slavik et al [34] McInnes and Hornmoen [35] Vos et al [36] Sutton et al [38] Young et al [40] Guidry et al [42] Renshaw et al [43] Vos et al [44]	12 (50)
	Instagram	Dimanlig-Cruz et al [31] Guidry et al [42]	2 (8)
	Other social media (Sina Weibo, TikTok, and YouTube)	Chen et al [26,30] Dimanlig-Cruz et al [31]	3 (13)
**Message framing**			
	Facebook	Jang and Baek [47]	1 (4)
	Twitter	Sutton et al [38] Pascual-Ferrá et al [45]	2 (8)
	Website	Ort and Fahr [46]	1 (4)
	Other social media (Kakao Talk)	Jang and Baek [47]	1 (4)
**Campaign effectiveness**			
	Facebook	MacKay et al [29] Duong et al [49]	2 (8)
	Website	Harris-Sagaribay et al [50]	1 (4)
	Other social media (YouTube and Zalo)	Duong et al [49]	1 (4)

#### Engagement

In 71% (17/24) of the studies, the primary aim was to assess the success of engagement techniques in web-based communication both on websites and social media.

First, what emerged in the study by MacKay et al [29] was that public health agencies and news media should use guiding principles consistently to increase positive sentiment and build trust among followers.

Another study by Alamoodi et al [12] was focused on TikTok with the aim of determining the factors and influencing mechanisms related to citizen engagement with the TikTok account of the National Health Commission of China during the COVID-19 pandemic. The result of this was that shorter videos are preferred to longer ones, and a positive emotion is better suited than a negative one. Similarly, a study carried out in China [30], this time on the platform Sina Weibo, concluded that posts displaying positive emotions can include more videos or pictures, whereas plain text is more suitable for posts with negative emotions.

The studies by Dimanlig-Cruz et al [31], Hagen et al [32], Lauran et al [33], and Slavik et al [34] dealt with targeting specific population groups. The first and most generalizable study is the one by Lauran et al [33], who stated that deciding on 1 actor and 1 (homogeneous) stakeholder group is not the right strategy. What is advisable is to take the perspectives of the multiple stakeholders into account (and find opinion leaders within those groups) when deciding on the communication strategies to use and to refrain from introducing a new, unrelated issue into the discussion before the original issue is handled. Similarly, according to Hagen et al [32], public health organizations can benefit from understanding the types of content that are transmitted through specific social media platforms and identifying key participants who are authoritative, popular, and connected with disparate communities to efficiently communicate with the public. As for the study by Dimanlig-Cruz et al [31], given the high number of youths on Instagram and YouTube, public health officials may want to consider targeting youths on these sites; similarly, Slavik et al [34] tried to assess tweeting practices during public health crises to improve risk communication and maximize engagement. What emerged was the need for public health agencies to monitor Twitter analytics to understand their audience and leverage whatever Twitter engagement strategies help maximize the shares of their communications.

Creating a community was also a very important point in these studies as coordinating communication efforts by frequently interacting with other organizations to boost one’s network position can facilitate further communication efforts [25]. In particular, what emerged is that organizations should consider retweeting content from health information sources with a high number of Twitter followers if they want to build up their own follower base and that health agencies should coordinate their communication efforts by frequently interacting with each other. This will boost their network position and facilitate further communication efforts. Another key strategy for public health agencies might be to develop a community of trusted users with their own significant base of followers who will pass on tweets from health authorities [35]. In the event of an outbreak, prompt responses from the authorities can be vital in crisis management, as explained by Vos et al [36], who stated that public health officials may want to emphasize the severity of an emerging infectious disease. Efficacy information is an important message element in encouraging an effective response. Precise guidelines have also been proposed in the event of a specific outbreak (COVID-19) or with regard to specific communication channels (Twitter). In the case of COVID-19, Lwin et al [37] focused on the dissemination of posts regarding the COVID-19 pandemic, and their findings showed that the public liked and shared the most in the preoutbreak phase and engaged with posts much less during the outbreak, as well as the fact that the public liked the most the posts that encouraged self-efficacy. Furthermore, in an uncertain environment, public agencies can reach the public—and increase message sharing—with a wide range of practical information regarding the health impacts of COVID-19, protective action measures, and the progress of the pandemic itself. At the same time, some tactics useful in other disasters (such as sentence styles that use exclamatory and interrogative punctuation) were counterproductive during the COVID-19 pandemic [38]. As for studies on Twitter, according to Tang et al [39], the main takeaway was that public health agencies should continue to use Twitter to disseminate information, promote action, and build communities, especially by targeting specific population groups. Similarly, Young et al [40] focused on chats, concluding that this means of communication was effective at answering questions about disease, creating a forum for targeted criticism, and promoting conversation among participants. Government accounts could also take full advantage of social media functions, especially mentions, hashtags, and the number of original posts, and add pictures and text length appropriately to increase interactions with the public and improve the level of engagement [41].

As for the strategies that proposed taking more technical aspects into account, the studies by Guidry et al [42], Renshaw et al [43], and Vos et al [44] offered interesting insights. First, according to Renshaw et al [43], focusing on useful content rather than gimmicks to go viral would be helpful in the long run. Having meaningful content such as relevant images embedded in posts might be crucial for success and, according to Guidry et al [42] and Vos et al [44], organizations should create messages that illustrate information visually and try to include threat and efficacy information in messages. They should also engage social media audiences before public health crises emerge.

#### Message Framing

This group comprises 17% (4/24) of the studies, all aimed at assessing the way in which a certain message is framed to make communication as effective as possible. To begin with, Pascual-Ferrá et al [45] concluded that the integration of social network analysis is recommended as a best practice in crisis communication on social media. Ort and Fahr [46] conducted a study focused on the interaction between perception of threat and self-efficacy in a crisis situation. Even in this case, health messages promoting people’s self-efficacy perceptions may be preferable to threatening messages. Another study aimed at a specific part of the population—public health officials—carried out by Jang and Baek [47] in South Korea concluded that lower perceived credibility of information from public health officials was associated with a greater tendency to use web-based news, interpersonal networks, and social media. The last study [48] focused on how message construction, style, content, and the textual content of tweets and embedded images affected message retransmission over the course of the pandemic. In particular, the focus was on tweet effectiveness, this time studying hashtags and concluding that public health–oriented hashtag campaigns may help engage individuals to help them feel part of a larger collective body and participate locally by contributing information about their local context.

#### Campaign Effectiveness

The last group in this category comprised 8% (2/24) of the studies, aimed at assessing the success of certain campaigns. First, Duong et al [49] investigated the content and format of physical distancing messages directed at Vietnamese youths during the COVID-19 pandemic, concluding that perceived norms and self-efficacy did not fully account for the association between interpersonal communication and behavioral intentions. Second, Harris-Sagaribay et al [50] summarized the lessons learned through an observational retrospective study when it comes to improving information dissemination during a health care crisis. Other than content, the effectiveness of website-based communication was measured through ease of navigation and trust in the information provided by the website.

### Studies on Disease Prevention and Health Promotion

#### Overview

This section comprises 45% (35/78) of the articles, all concerning studies that deal with the themes of health promotion and disease prevention (Table 2). Of these 35 studies, 19 (54%) were carried out in the United States, 5 (14%) were carried out in Australia, and 4 (11%) were carried out in the United Kingdom. Most studies had to do with engagement (19/35, 54%), 23% (8/35) had to do with campaign effectiveness strategies and, finally, 23% (8/35) had to do with message framing. The Kmet evaluation resulted in an average of 0.77 points, with a correlation coefficient of 0.78.

Studies on these 2 topics were mostly based on the analysis of collections of posts (21/35, 60%) rather than being studies on groups of people (14/35, 40%). The study designs were also diversified in this group, with the most represented being content analyses (16/35, 46%) and observational studies (9/35, 26%). In this group, most studies (27/35, 77%) were conducted on only 1 communication medium, whereas the remaining 23% (8/35) dealt with multiple platforms. As for communication theories, 71% (25/35) of the studies referred to one or more specific communication theories, and 29% (10/35) did not.

**Table 2:** List of studies on disease prevention and health promotion (see Multimedia Appendix 1 for more details; N=35).

Primary evaluated aspect and communication medium		Reference	Studies, n (%)
**Engagement**			
	Facebook	Alonso-Cañadas et al [51] Loft et al [52] Syred et al [53] Zhang and Zhou [54] Kite et al [56] Lister et al [57] Parackal et al [58] Reuter et al [60] Barklamb et al [61] Klassen et al [63] Rus and Cameron [66] Strekalova and Krieger [67] Theiss et al [68]	13 (37)
	Twitter	Zhang et al [55] Lister et al [57] Rabarison et al [59] Reuter et al [60] Kim et al [62] Guidry et al [64] Chung [65]	7 (20)
	Instagram	Reuter et al [60] Barklamb et al [61] Klassen et al [63] Alkazemi et al [69]	4 (11)
	Website	Lister et al [57]	1 (3)
	Other social media (anonymous discussion platform)	Zhang et al [55]	1 (3)
**Message framing**			
	Facebook	Dockter et al [24] Borah and Xiao [28] Yoo et al [70] Parackal et al [72]	4 (11)
	Twitter	Yoo et al [70] Cho et al [71] Chung and Lim [74]	2 (6)
	Instagram	Yoo et al [70] Nobles et al [73]	2 (6)
	Website	Chung and Lim [74] Whitten et al [75]	3 (9)
	Other social media (YouTube, Flickr, Kakao Story, and Naver Band)	Yoo et al [70]	1 (3)
**Campaign effectiveness**			
	Facebook	Potente et al [27]	1 (3)
	Twitter	Allen et al [77] Yoo et al [79]	2 (6)
	Website	Harris et al [76] Frisch et al [78] Nguyen et al [80] Perrault and Silk [81]	5 (14)
	Other social media (YouTube and Myspace)	Potente et al [27]	1 (3)

#### Engagement

In this group, 54% (19/35) of the studies analyzed the engagement of campaigns or interventions aimed at promoting disease prevention or health promotion measures.

One of the main takeaways from these studies is that the message format affects the level of users’ web-based commitment to health organizations via social media [51]. Other studies (2/35, 6%) were tied to the assessment of the effectiveness of different types of posts on Facebook. A study analyzed the extent to which a post can resonate with an indecisive parent when it comes to the human papillomavirus vaccine [52], concluding that designing factual posts so that they include an emotional dimension increased the engagement with these posts, sponsored content can generate more negative comments than organic content, and all people should be addressed in an accommodating manner regardless of their tone. Although Syred et al [53] stated that moderation can help maintain the discussion quality and generate new interest and discussion on a certain topic, Loft et al [52], by contrast, focused more on the technical aspects of Facebook posts by stating that photos with short comments were the most effective in engaging information consumers and greater use of this post type could encourage greater audience engagement. At the same time, professional videos may not be as effective as a mechanism for active audience engagement on social media platforms. Zhang and Zhou [54] analyzed message efficacy, this time with particular attention to fear, and proposed a strategy where the inclusion of more emotional cues such as pictures is emphasized to arouse fear to motivate information dissemination on social media. In the social media context (in this case, Twitter and anonymous web-based discussion platforms), the relative importance of having clear informational content sent from organizations would be much greater for generating highly viewed and shared cancer prevention messages [55].

The studies by Kite et al [56], Lister et al [57], Parackal et al [58], Rabarison et al [59], and Reuter et al [60] measured engagement in and of itself. In particular, the study by Rabarison et al [59] focused on a specific aspect of the social media in question: Twitter chats. Specifically, chats of this kind should be used as an engagement tool with the audience by sharing messages and responding to questions from the public. Focusing on Twitter, Instagram, and Facebook, Reuter et al [60] concluded that engagement with a health message on social media does not indicate user engagement on a website and, therefore, it is recommended that both metrics be taken into account when designing health promotion strategies. It was also suggested to combine organic and advertising messages in health promotion campaigns. More specifically, with regard to Facebook, communication effectiveness could be enhanced using a two-way communication format, which enables the promoter to respond to negative comments [58]. Finally, according to the studies by Kite et al [56] and Lister et al [57], effective engagement through Facebook requires both maximizing the reach of posts through paid boosts and delivering content that users want to engage with and share to capitalize on word-of-mouth marketing.

In total, 6% (2/35) of the studies suggested social media strategies with the aim of improving engagement by comparing the work of institutional social media with that of lifestyle influencers [61] or by investigating the way in which network structures explain retweeting behaviors [62]. More specifically, Klassen et al [63] and Kim et al [62] stated, respectively, that health promotion organizations should try to build relationships with their users in a similar fashion to lifestyle brands and that influential people should be identified and targeted as their messages are more likely to be disseminated.

Regarding more technical aspects, the study by Guidry et al [64] states that, in the case of crisis communication, public health organizations should be present on all major social media platforms, but Instagram may yield the greatest return and user engagement. The study by Chung [65] was aimed at examining whether dialogic messages induced greater risk-preventive behavioral intentions than monologic messages, reaching the conclusion that frequent posting of tweets with images and graphs instead of videos and hyperlinks is beneficial. Similarly, according to Barklamb et al [61], strategies that were associated with higher engagement included the use of hashtags and announcements compared with not prompting engagement strategies. However, imagery should be carefully used as it appeared to be a powerful tool for attracting attention and briefly engaging users (ie, increasing likes) as well as increasing message transmission (ie, increasing shares). However, the use of images with information about illness consequences and control or with messages conveying negative affect could mute responses [66]. In particular, communication effectiveness could also be enhanced by designing factual posts so that they include an emotional dimension that could increase engagement [67]. Moreover, according to Strekalova and Krieger [67], sponsored content can generate more negative comments than organic content, and all people should be addressed in an accommodating manner regardless of their tone. Finally, users were more likely to click, share, comment, or like the content of posts that had photos. Branded, visual content was more effective in facilitating engagement [68].

Finally, 3% (1/35) of the studies focused on the effectiveness of communication theories, in particular that of the health belief model. Analyzing the Instagram accounts of the health departments of the Gulf Cooperation Council, it was found that the health belief model should be included more in internet-based communication [69].

#### Message Framing

Considerably less studies (8/35, 23%) dealt with the topic of message framing in this category. The first study in this category was by Dockter et al [24], stating that content should be transmitted or retransmitted by well-known, credible sources. On a more specific note relating to content engagement, Yoo et al [70] recommended the use of content-oriented social media when trying to influence risk perception during campaigns, with particular attention to posts with photos as users were more likely to click, share, comment, or like this type of content. Borah and Xiao [28] and Cho et al [71] investigated the effect of health message framing and the moderating effects of social endorsement and source type on credibility perceptions of posts, resulting in a superiority of gain-framed messages to reach a positive campaign outcome. Other studies (4/35, 11%) were tied to the assessment of the effectiveness of different types of posts on Facebook when it comes to engagement [72]. In particular, communication effectiveness could be enhanced using a two-way communication format, which enables the promoter to respond to negative comments. Nobles et al [73] examined the demographic profile in photos concerning HIV prevention and diagnosis, underlining a disparity in the representation of minorities and marginalized communities. Another study by Chung and Lim [74] focused on a long-running campaign on National Breast Cancer Awareness Month and concluded with 2 observations regarding the efficacy of frequent posting and the positive impact of photos and images instead of videos and hyperlinks. Finally, Whitten et al [75] addressed the presence of information targeting low-literacy, racially diverse, non–English-speaking, and age-diverse audiences on breast cancer websites. The results were three-fold: if content were tagged according to ethnicity or language, then this would allow users to browse websites according to the information that is most personally relevant; it would be beneficial for websites containing lower-literacy material to avoid statistical data; and storytelling evidence has demonstrated the ability to serve as a greater motivator for healthy behaviors.

#### Campaign Effectiveness

This last group included 23% (8/35) of the studies, which focused on the assessment of the success of a particular campaign. Starting again from a more general framework, the study by Harris et al [76] stated that there are 4 qualities that are key to influencing trust and the subsequent decision to act on the advice given. These are information quality, personalization, perceived impartiality, and design credibility. Delving deeply into the issue of trust, the studies described in this section proposed different strategies to maximize trust from the web users. Social media can also be used effectively in social marketing campaigns and is an essential tool in the promotional mix when targeting young people. According to Potente et al [27], entertaining peer-to-peer messages can be used to engage youths with an important health message for skin cancer prevention. By contrast, Allen et al [77], on the promotion of the human papillomavirus vaccine, recorded no statistically significant change in the intent to be vaccinated in the next 6 or 12 months after the campaign among those who had not yet started or completed vaccination. Focusing on more technical aspects, Frisch et al [78] stated that websites designed for health education should include visual presentations of information such as pictures, charts, or graphs. Similarly, Yoo et al [79] were concerned with more technical aspects of Twitter communication, especially when developing a Twitter campaign. The results included the need to consider incorporating features such as hyperlinks to related websites or live chats with health care providers as well as the creation of tailored messages or edutainment, which may also be considered to engage people in the process of information selection and transmission. Moving forward from just design structure, the way content is presented is also a great source of studies. In this case, Nguyen et al [80] offered useful insights, concluding that mode tailoring may be a tool to reduce or prevent the information overload that may occur when too much information is placed on a nontailored web page at one time. Other than content, the effectiveness of website-based communication is measured through ease of navigation and trust in the information provided by the website. Perrault and Silk [81] used social cognitive theory and media richness theory to prove that the exposure to videos was responsible for the increased engagement in risk-reduction behaviors. Another communication theory is the transtheoretical model. This was used in the study by Pirzadeh et al [82], who stated that the transtheoretical model was the most effective education strategy when it comes to prompting behavior change.

### Studies on General Health

#### Overview

In the category of general health (Table 3), 17% (13/78) of the studies were included. Of these 13 studies, 9 (69%) were carried out in the United States, whereas 3 (23%) were carried out in the United Kingdom. Engagement was, once again, the most represented topic (9/13, 69%), followed by message framing (4/13, 31%). The same proportion holds true when it comes to studies on collections of posts (9/13, 69%) versus studies on human samples (4/13, 31%). The study designs were not overly diversified in this group as 46% (6/13) were observational studies and 38% (5/13) were content analyses. In this group, all studies (13/13, 100%) were conducted on a single communication medium, but only 31% (4/13) were connected with a specific communication theory (the remaining 9/13, 69% were not). The Kmet evaluation resulted in an average of 0.76 points, with a correlation coefficient of 0.82.

**Table 3:** List of studies on general health (see Multimedia Appendix 1 for more details; N=13).

Primary evaluated aspect and communication medium			Reference		Studies, n (%)
**Engagement**					
	Facebook	Bhattacharya et al [86] Kite et al [87] Campbell and Rudan [88]		3 (23)	
	Twitter	Bhattacharya et al [83] Fung et al [84] Meng et al [85] Park et al [90]		4 (31)	
	Instagram	Kim and Kim [89]		1 (8)	
	Website	Pang et al [91] Hung and Stones [92] Lazard and Mackert [93] Shim and Jo [94] Sillence et al [95]		5 (38)	

#### Engagement

In this group, 15% (2/13) of the studies [83,84] focused on the content of health organizations’ Twitter profiles, concluding that the use of hashtags, URLs, visual cues, and user mentions was positively associated with retweets. Another study focusing on retweets and on the type of message brought on by health organizations is the one by Meng et al [85]. They defined a series of recommendations on the matter (ie, designing efficacious information is the key to increasing the aggregated number of retweets, crafting information that can raise risk perception is important to increase the diffusion chain through person-to-person transmission, and tweets that induce negative emotions could be more effective in catching users’ attention and expanding sharing of the information). A similar study by Bhattacharya et al [86], this time on Facebook, also stated that posts containing media or links and expressing positive sentiments correlated with higher or longer engagement. Facebook was also the topic of research of Kite et al [87], who concluded that content providers must encourage engagement and adapt to the Facebook algorithm to maximize message exposure while also ensuring that the content is of high quality. Language also plays an important role in the effectiveness of a post, as explained by Campbell and Rudan [88], who claimed that adjusting the language and presentation can be of more appeal to popular culture. Kim and Kim [89], by contrast, centered their study on the Instagram presence of the Centers for Disease Control and Prevention (CDC), stating that the message design should be different depending on whether the aim is to increase the number of likes and comments or induce a more positive response from the public. The dissemination of health information is also a topical area of research, in particular by Park et al [90], who provided guidelines such as retweeting content from health information sources with a high number of Twitter followers to build up an organization’s follower base. The study by Pang et al [91] was aimed at creating a design for a consumer health website by supporting different health-seeking behaviors. In particular, different types of information-seeking behavior should be supported as access to a dynamic information scope is critical for health information seeking.

#### Message Framing

This group comprised 4 studies: the studies by Hung and Stones [92], Lazard and Mackert [93], Shim and Jo [94], and Sillence et al [95].

Concerning design and website presentation, Lazard and Mackert [93] stated that high design complexity is often associated with a better perceived comprehensibility, a greater perceived usefulness, a greater message quality, and higher perceptions of visual informativeness. Other than content, the effectiveness of website-based communication is measured through ease of navigation and trust in the information provided by the website. Sillence et al [95] identified the key factors influencing UK and US citizens’ trust and intention to act on advice found on health websites (ie, credibility and impartiality). Moving forward from just design structure, the way content is presented is also a great source of studies. In this case, Hung and Stones [92] offered useful insights, stating that, among other guidelines, to appeal to the public, general terms should substitute professional terms and simplified text-based content should be used. Delving deeply into the issue of trust, Shim and Jo [94] applied the information systems success model, assessing that service quality had a significant association with user satisfaction and that its impact on perceived benefits occurred indirectly to user satisfaction and intention, thus maximizing trust from the web users.

### Health Literacy and Misinformation Correction

This is the smallest group in this corpus (Table 4), with only 8% (6/78) of the studies. They are divided into 2 categories: misinformation correction (4/6, 67%) and health literacy (2/6, 33%). Given the scarcity of examples of these types of interventions, they were grouped together. Of these 6 studies, 4 (67%) were carried out in the United States. Contrary to the other groups, studies focused on the analysis of engagement were not present in this group, and only message framing and campaign effectiveness were represented. All the studies (6/6, 100%) had groups of participants as their main sample. All the studies in this group (6/6, 100%) referred to a single communication medium. Of the 6 studies, 1 (17%) did not present a reference to a specific communication theory. The Kmet evaluation resulted in an average of 0.80 points, with a correlation coefficient of 0.59.

The misinformation correction studies were those by Bode and Vraga [96], Gesser-Edelsburg et al [97], and Vraga and Bode [98,99]. First, Bode and Vraga [96] stated that corrective information provided via an algorithm or social connections reduces misinformation and is effective as they are considered credible, whereas Gesser-Edelsburg et al [97] stated that it is important for organizations to correct misinformation transparently while at the same time addressing the emotional aspects that may come into play in case of conflicts of opinion. The study by Vraga and Bode [99] was carried out to test the efficacy of shareable infographics to debunk COVID-19 myths. In particular, one of the solutions found by the authors was that organizations can debunk misinformation circulating in society by sharing high-quality information on social media, emphasizing the facts without waiting to see them shared directly in their feeds, which expands the opportunities for observational correction to occur. Information correction is an area of interest that is also reported in this group, with the study by Vraga and Bode [98] testing whether the number and source (user vs the CDC) of corrective responses affect the successful reduction of misperceptions. Thus, this study suggests that organizations should speak up when they see misinformation on social media and reputable organizations such as the CDC should monitor social media feeds and immediately attempt to rebut misinformation when it arises.

The last 33% (2/6) of the studies dealt with health literacy and were all designed to develop different approaches aimed at different categories. The first of these studies is the one by Chin et al [100], which focused on older adults and proposed a multifaceted approach guided by theories of text comprehension and document design to improve readability for websites, in particular claiming that increasing document readability alone is insufficient for improving text comprehension in older adults. Meppelink et al [101] added to this statement by suggesting the use of a visual representation of information to improve the retention of information.

**Table 4:** List of studies on misinformation correction and health literacy (see Multimedia Appendix 1 for more details; N=6).

Primary evaluated aspect and communication medium			Reference		Studies, n (%)
**Message framing**					
	Facebook	Gesser-Edelsburg et al [97] Vraga and Bode [99]		2 (33)	
	Website	Chin et al [100]		1 (17)	
**Campaign effectiveness**					
	Facebook	Bode and Vraga [96]		1 (17)	
	Twitter	Vraga and Bode [98]		1 (17)	
	Website	Meppelink et al [101]		1 (17)	

### Quality Assessment

The Kmet evaluation was used to distinguish between studies presenting a solid structure and studies lacking those factors, as made explicit by the low score obtained for the Kmet evaluation. In particular, considering the 0.75-point threshold, 36% (28/78) of the studies were excluded. To be more specific, of the 78 studies, 3 (4%) had <0.55 points, 25 (32%) were between the 0.55- and the 0.75-point mark, and 50 (64%) were above the 0.75-point threshold.

Table 5 shows the distribution of the studies’ quality for each research category using the 0.75-point mark as a threshold. The health promotion and disease prevention category had a higher percentage of good-quality studies than the other categories (*P*=.02).

**Table 5:** Research categories and distribution of quality studies according to the 0.75-point Kmet score threshold (N=78).

	Studies below cutoff, n (%)	Studies above cutoff, n (%)
Crisis communication (n=24)	13 (54)	11 (46)
Health promotion and disease prevention (n=35)	10 (29)	25 (71)
General health (n=13)	9 (69)	4 (31)
Misinformation correction and health literacy (n=6)	1 (17)	5 (83)

## Discussion

### Principal Findings

Our review provides insights into topics regarding the different modes of communication used by health care authorities to engage with the public in different situations, namely, crisis communication and health promotion and disease prevention. Previous systematic reviews have dealt with this topic by focusing on certain specific aspects such as communication effectiveness for specific channels and situations. This systematic review aimed to provide a more comprehensive view of internet-based health communication. The amount of works included in this study also suggests a high interest in this particular topic. It is also worth mentioning that health communication represents a vital point for public health as the rapid diffusion of information to the largest possible number of users is key when trying to effectively communicate important information, as also recently seen during the COVID-19 pandemic.

The results of this systematic review raise an important question: is it possible to define a series of key points to address the basics of internet-based communication for public health?

**Figure 2:**
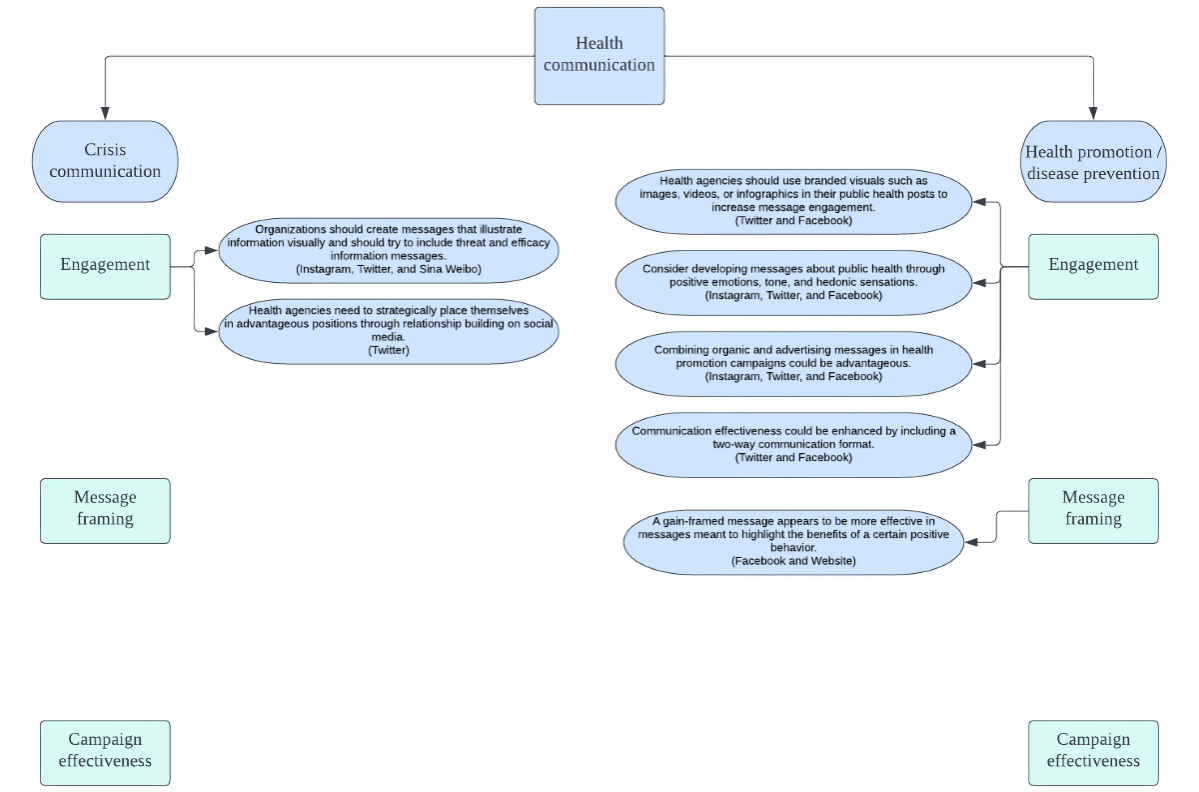
Flowchart of key points.

To do so, a flowchart (Figure 2) was created, starting from the basic distinction between the 2 main themes that are addressed by the selected studies: crisis communication and health promotion and disease prevention. The other 2 categories identified in this review (general health and health literacy and misinformation correction) were not considered as they were of too general or too narrow scope to constitute a relevant sample. This distinction was made necessary as these 2 types of communication account for 2 almost opposite situations and purposes.

Going further into this analysis, it was vital to make a distinction between the different primary evaluated aspects (ie, engagement, message framing, and campaign effectiveness). This was done because the conventions and communication strategies used differed greatly, especially given the different nature of communication for those purposes. An important point to be made regards the criteria according to which the key points were chosen to be included in this flowchart. A first measure is represented by the Kmet score of the study. As this score is used to address the overall soundness of the research process, only the studies that recorded a score ≥0.75 points were taken into account. Another measure included was the repetition of certain suggested recommendations or conclusions throughout the group in question. What this entails is that a certain specific proposed strategy that was repeated at least two times was included in the final flowchart as it can be assumed that it was more easily applicable in a more general context. Figure 2 offers a more thorough rundown of the included key points identified in this systematic review. As we can see, not all the primary evaluated aspects are paired with one or more key points as, in some cases, the studies in question did not meet the selected criteria. As for crisis communication, only the engagement category presented 2 key points: one regarding the need to create messages that visually convey information and the other addressing the need for health agencies to place themselves in advantageous positions when it comes to relationship building on social media. As for health promotion and disease prevention, engagement recorded 4 key points having to do with creating effective visual information, promoting the use of a positive tone in messages, combining organic and advertising messages, and implementing a two-way communication. One last indication was made for message framing, underlying the greater effectiveness of gain-framed communication.

To be noted is also the fact that the key points proposed are not universally applicable to all communication channels but, rather, to specific ones. The proposed flowchart includes indications as to which communication channel the proposed key points are applicable to (ie, websites, Facebook, Twitter, Instagram, and Sina Weibo).

This systematic review met the criteria of Assessing the Methodological Quality of Systematic Reviews [102], a measurement tool to assess the methodological quality of systematic reviews, thus ensuring the accuracy of the reviewed data. However, some limitations should be addressed.

### Limitations

Through a systematic approach, we aimed to provide a comprehensive look at health care communication via different media and with different purposes. Although the number of articles retrieved was relatively large, some limitations related to the study design must be underlined. As already mentioned, the large number of studies not meeting the threshold (28/78, 36%) suggests a lack of soundness of the selected studies, thus calling attention to a need for more in-depth research on the topic of internet-based communication as well as measures for campaign and intervention effectiveness.

This need is also tied to another limitation of this study, posed this time by the lack of appropriate measures to evaluate the quality of the studies of this sort. In this case, the Kmet evaluation tool, albeit flawed, represented the best possible measure to evaluate studies on health communication. However, this tool is directed at the assessment of primary research reports in the field of medical experimental research, and a number of the areas of evaluation were impossible to relate to these studies (ie, randomization, double-blind, sample size appropriateness, and control for confounding). As the scale for evaluation appears to be rather limited, the scores attributed to the studies in this systematic review were based on an average of circa 18 maximum points against an actual maximum of 24 points. Therefore, this raises the need for a more precise tool to evaluate this type of studies.

Another issue lies in the fact that the studies taken into account for this systematic review do not have a uniform end point as, for example, some focus on the reach of a campaign or on user behaviors on social media. This results in a multitude of different measures of success that make it difficult to properly understand and evaluate the reach and success of a campaign or of certain web-based behaviors. This variety in end points is also reflected in the variety—or lack thereof—of measures of success of a certain campaign or policy. Thus, it is difficult to assess a baseline measure of effectiveness for each of the communication channels described in this review, which points to the necessity of forming a medium-specific criterion for this kind of evaluation.

A final limitation is related to the extreme specificity of web-based communication based on geographical as well as cultural differences that make it difficult to form a comprehensive list of guidelines for this type of discourse. The use of the internet and social media and the strategies and practices adopted by single countries or even smaller cultural groups is an issue so big that it is impossible to look away from it when considering this type of studies, which makes it difficult to redact a list of guidelines to adopt when trying to manage internet-based health communication. Thus, this creates the need to always address the country of origin of a study as well as its specific target audience.

### Conclusions

The evidence gathered in this study suggests that no single strategy works best in the case of health care communication. Although there is evidence supporting multiple communication approaches across different media, how the interaction unfolds must be resolved according to a number of variables: communication medium (website or social media), country of intervention, organization type (health organization or health ministry), and aim of the intervention.

This extreme variability of outcomes and the lack of a unitary measure for assessing the end points of a specific campaign or study on individuals lies in the inherently fluid and ever-changing essence of communication practices, which makes it difficult to define this concept altogether as well as grasp a precise definition of what *evidence* entails in this field compared, for example, with the evidence gathered through randomized controlled trials and cohort studies in the medical field.

In practice, this review tried to provide a baseline for practitioners and researchers as to how to conduct a campaign on the web on different web-based communication channels. However, as stated before, this is not enough to provide a comprehensive set of guidelines on the matter; rather, as a matter of fact, it raises more questions that need to be addressed in future research, in particular on the matter of forming a unified measure of effectiveness for campaigns and policies and on the scales used to evaluate the soundness of a certain study.

## Appendix

Multimedia Appendix 1 Extended tables describing the studies collected for this research.

## Abbreviations

CDC: Centers for Disease Control and Prevention
PRISMA: Preferred Reporting Items for Systematic Reviews and Meta-Analyses
