# Supplementary material for: Evaluating the Effectiveness of Internet-Based Communication for Public Health: Systematic Review
Source: J Med Internet Res. 2022 Sep 13;24(9):e38541. doi: 10.2196/38541 (PMC9516364; doi:10.2196/38541)
Supplement: Multimedia Appendix 1 [file jmir_v24i9e38541_app1.pdf]

Table S1. List of studies on crisis communication

References	Year	Country	Aim	Communication channel	Theories or models used (if any)	Study design	Sample	Observation time	Primary evaluated aspect	Main results	Proposed Strategy / Practical Implication	KMET
Chen *et al.*	2021	CN	Determine the factors and influencing mechanisms related to citizen engagement with the TikTok account of the National Health Commission of China during the COVID- 19 pandemic.	TikTok	n/a	Observational	Posts (335)	3 months (21 January 2020 - 25 April 2020)	Engagement	• Shorter videos and videos with longer titles generate greater engagement<br>• Higher levels of dialogic loop significantly reduce the number of shares received on TikTok<br>• Longer videos with positive titles receive higher numbers of likes and comments<br>• Longer videos with negative titles receive lower numbers of likes and comments	• Shorter video length is to be preferred<br>• If the video length is relatively long, it is better to have a title with positive emotion rather than negative emotion	0.72
Chen *et al.*	2020	CN	Examine how citizen engagement relates to a series of theoretically relevant factors, including media richness, dialogic loop, content type, and emotional valence.	Sina Weibo	Media Richness Theory (MRT)<br>Dialogic Communication Theory (DCT)	Observational	Posts (1,411)	51 days (14 January 2020 - 5 March 2020)	Engagement	• Media richness was negatively associated with citizen engagement<br>• This study confirms that the dialogic loop will increase citizen engagement during public health crises<br>• Emotional valence will moderate the effects of media richness, dialogic loop, and content type (latest news) on citizen engagement	• Government agencies should actively use the dialogic loop to enhance citizen engagement<br>• Those posts displaying positive emotions can include more videos or pictures, while plain text is more suitable for posts with negative emotions	0.78
Dimaling-Cruz *et al.*	2021	CA	Assess the content and format of phisical distancing messaging targeted at youth and young adults aged 16–29 years across social media platforms on accounts belonging to Canadian public health entities (PHEs); examine the use of BCTs for PD-related social media posts that implicitly and explicitly target youth.	Facebook<br>Instagram<br>Twitter<br>YouTube	Behavioural Changing Techniques	Content analysis	Posts (319)	2 months (1 April - 31 May 2020)	Engagement	The majority of posts were implicitly targeting youth, with only 5.3% of the posts explicitly mentioning them. The most interactive implicitly targeted posts consistently resulted in higher numbers of interactions compared to the most interactive explicitly targeted posts, regardless of social media platform.	Many of the PHE accounts used the same post multiple times on different days. This may be seen as either an advantage or a disadvantage; Given the high number of youth on Instagram, PHEs may want to consider targeting youth on this site; Given the high concentration of youth on YouTube and the amount of time spent on this platform, PHEs may want to consider using YouTube in future health promotion campaigns in order to target the audiences 18 and younger.	0.83
Duong *et al.*	2021	VN	Evaluation of the success story of Vietnam's ability to control the COVID-19 outbreak in the early stages to examine the associations between exposure to the Vietnam Ministry of Health's COVID-19 prevention social media campaign messages, interpersonal communication, attitudes, perceived norms, self-efficacy, and intentions to stay at home.	Facebook<br>YouTube<br>Zalo	Integrative Model of Behavoiural Prediction	Observational	Adults (360)	2 weeks (1 April - 15 April 2020)	Campaign effectiveness	• Results showed that participants viewed the MoH's COVID-19 prevention social media messages and talked to others about the disease<br>• Results also revealed that interpersonal communication shaped attitudes, injunctive norms, descriptive norms, and self-efficacy	It was suspected that participants were aware of the benefits of the staying at home and thus formed highly favorable attitudes toward stay ing at home behavior. Results also further revealed that perceived norms and self-efficacy did not fully account for the association between interpersonal communication and behavioural intentions.	0.86
Guidry	2017	US	Employ a content analysis to examine how leading health-focused organizations utilized Twitter and Instagram in Ebola communications to their external publics.	Twitter<br>Instagran	n/a	Content analysis	Tweets (779)	3 months (September 2014 - December 2014)	Engagement	• The highest levels of public engagement were elicited by posts sent by MSF while using Instagram<br>• MSF used more photos, mention,s and hyperlinks	• Public health organizations should be present on all major social media platforms but Instagram may yeld the greates return<br>• Health organizations should utilize photos in risk-based communication on social media, even on non-visual platforms<br>• Strategic use of risk communication principles appears to be associated with greater public	0.75

											engagement and should be considered by public health organizations when designing future messaging and communication campaigns	
Hagen *et al.*	2018	US	Conduct a content and network analyses on a sample of Zika-related tweets regardig the 2015-2016 Zika outbreak in the US.	Twitter	n/a	Content analysis	Tweets (359,043)	11 days (25 August 2016 - 5 September 2016)	Engagement	• Government institutions such as the White House, CDC, and HHSGov were found to be highly authoritative and trustworthy sources<br>• Entities with the highest betweenness centrality are mostly experts in infectious disease, several actors involved in politics also have high betweenness centrality	Public health organizations can benefit by understanding the kinds of content that is transmitted through specific social media platforms and by identifying key participants who are authoritative, popular, and connected to disparate communities in order to efficiently communicate with the public.	0.64
Harris-Sagaribay *et al.*	2020	US	Summarise partnership activities, common inquiries, and lessons learned to improve partnerships and information dissemination during a future public health emergency.	Website	n/a	Observational	Inquiries across platforms (5387)	3 years (June 2016 - June 2019)	Campaign effectiveness	MotherToBaby received 5,387 Zika related inquiries from the public and healthcare providers between June 2016 and June 2019.	• Making use of the expertise and infrastructure of existing organizations saves critical time in responding to public health threats<br>• Referring the public to expert groups that individuals can contact directly, expands the ways in which consumers can access information<br>• Cross-referencing official guidance across organizations extends the reach of the content and demonstrates a consensus from trusted public health organizations<br>• Establishing a means early on to collect detailed data on public inquiries allows organizations to measure their impact and adapt messaging in response to areas of public concern or confusion	0.46
Jang *et al.*	2019	KR	Examine how credibility of information from PHOs is associated with the use of particular communication channels among a wide array of alternatives.	Television<br>Newspapers<br>Facebook<br>KakaoTalk	Media Dependency Theory<br>Source Credibility Research	Observational	Adults (1036)	June 15 and June 21, 2015	Message framing	Lower perceived credibility of information from PHOs was associated with a greater tendency to use onlinenews, interpersonal networks, and social media.	To control the diffusion of misinformation, PHOs need to disclose trustworthy information promptly and preemptively. PHOs'prompt and preemptive information management helps people obtain accurate information and develop appropriate risk sensing.	0.67
Kim *et al*.	2021	US	Explore how health agencies' network positions may affect their public engagement outcomes.	Twitter	n/a	Network analysis	Public Health organizations (132)	4 months (21 January 2020 - 31 May 2020)	Engagement	Health agencies that perform the role of information authority (high authority centralities) and relationship brokers (high brokerage scores) tend to receive significantly higher levels of public engagement than their counterparts.	• Health agencies need to strategically position themselves in advantageous network positions through relationship building on social media<br>• Public health agencies should coordinate their communication efforts by frequently interacting with each other. This will boost their network position and facilitate further communication efforts	0.81
Lauran *et al.*	2020	NL	Analyse whether and how different groups of stakeholders frame a crisis and the extent to which they attribute responsibility for the crisis to actors. The crisis in question is the contamination of eggs with Fipronil in the Netherlands.	Twitter	Situational Crisis Communication Theory (SCCT)	Content analysis	Tweets Network analysis (87,454)<br>Tweets content analysis (1,373)	80 days (17 July 2017 - 5 October 2017)	Engagement	• It is possible to distinguish between different groups within the network of authors tweeting about fipronil<br>• Different groups attribute responsibility differently in terms of frequency, type and actor<br>• Opinion leaders attribute victim responsibility more often, while less influential people attribute accidental or intentional responsibility more often<br>• Different groups within this network use different frames to address the problem of the crisis	• Deciding upon one actor and one (homogeneous) stakeholder group is not the right strategy<br>• It is advised to take the perspectives of the multiple stakeholders into account when deciding upon the communication strategies to use and to refrain from introducing a new, unrelated, issue in the discussion before the original issue is handled<br>• Finding opinion leaders in a group and communicating with them to disperse a message throughout the group might be more effective than communicating to the group as a whole	0.53

Lwin *et al.*	2018	SG	Analyse how social media may be utilized to implement and adapt the CERC model.	Facebook	Crisis and Emergency Risk Communication Model (CERC)	Content analysis	Articles (51)	1 year (January 2016 - December 2016)	Engagement	• Findings showed that the public liked and shared the most in the pre-outbreak phase, and engaged much less in the outbreak messages than they did in the pre-outbreak phase<br>• The public liked posts the most that encouraged self-efficacy	Pre-outbreak messages on social media should include all the necessary information to update and educate the public about the risk of the potential crises, while providing reassurance and self-efficacy.	0.67
MacKay *et al.*	2021	CA	• Develop a succinct list of guiding principles for crisis communication on social media;<br>• Analyze whether and how Canadian public health and news media are incorporating these guiding principles into their COVID-19 crisis communication on Facebook;<br>• Determine the topic(s) of COVID-19 crisis communication on Facebook;<br>• Understand public sentiment within comments in relationship to the guiding princi ples and topics included in COVID-19 crisis communication on Facebook;<br>• Examine the relationship between the guiding principles used in COVID-19 crisis communication Facebook posts and the corresponding sentiment by source.	Facebook	n/a	Content analysis	Posts (438)	6 months (31 december 2019 - 14 June 2020)	Campaign effectiveness	The guiding principles for effective crisis communication were inconsistently applied and combined. A limited combination of guiding principles, especially those that demonstrate trustworthiness, was likely driving the negative sentiment uncovered in the comments.	Public health and news media should use the guiding principles consistently to increase positive sentiment and build trust among followers.	0.67
McInnes *et al.*	2018	UK<br>NO	Examine the policies and strategies Norway and the UK had established by the time of the Ebola outbreak for the use of social media during health emergencies.	Twitter	n/a	Interviews<br>Content analysis	Tweets (42)	n/a	Engagement	• Traditional paradigms of crisis communication persist in the social media environment<br>• Authorities have adapted to using social media as a monitoring tool, but not to its dialogical nature<br>• Twitter appears better geared to respond to individual events than to long wave events such as a disease outbreak	A key strategy for public health agencies might be to develop a community of trusted users with their own significant base of followers who will pass on tweets from health authorities.	0.72
Ort *et al.*	2018	CH	Investigate the effect of invoked fear via health communication messages on crucial components of the Extended Parallel Process Model (EPPM) by focusing on the rarely examined interaction between perceptions of threat and efficacy and their effects on fear control and danger control processes as well as health-relevant outcomes.	Website	Extended Parallel Process Model (EPPM)<br>Theory of Planned Behavior (TPB)	Experimental	Adults (447)	n/a	Message framing	• Efficacy plays a more important role than threats in convincing people to adopt health-related behaviours<br>• Threat to freedom led to a more negative attitude towards the vaccination whereas a positive message judgement was positively associated with favourable attitudes	Health messages promoting peoples' self efficacy perceptions may be preferable to threatening messages.	0.82
Pascual-Ferra *et al.*	2020	US	Demonstrate the use of social network analysis to understand public discourse on Twitter around the novel coronavirus disease 2019 (COVID-19) pandemic.	Twitter	n/a	Network analysis	Tweets (300,000)	3 days (30 January 2020, 11 March 2020, 8 June 2020)	Message framing	• Network of conversations around COVID-19 is highly decentralized, fragmented, and loosely connected<br>• Competing conversations, misinformation, and other distractions by politically motivated actors can hamper risk communication efforts by public health officials and health agencies	The integration of SNA is recommended as a best practice in risk communication on social media	0.68
Renshaw *et al.*	2021	US	Investigate how message construction, style, content, and the textual content of embedded images impacted message retransmission over the course of the first 8 months of the COVID-19 pandemic in the United States.	Twitter	n/a	Content analysis	Tweets (372,466)	8 months (1 January 2020 - 31 August 2020)	Engagement	• Including a video in a tweet was found to increase retransmission of the associated message by 24%. Quote tweets increased retransmission by 8%<br>• The inclusion of question marks (11%), exclamation marks (17%), user mentions (20%) hyperlinks/URL (32%) and especially replies (82%) decreased the rate of message retransmission<br>• Images containing text relating to surveillance key- words, information about susceptibility, and efficacy had the greatest positive impacts on retransmission (25%, 20%, and 18%, respectively)	• Focusing on useful content rather than gimmicks to go ''viral'' will be helpful in the long run<br>• Having meaningful content embedded in the images is crucial for their success—adding images without relevant content can potentially reduce message retransmission<br>• Timing messages to hit during this active time periods (the weekend during the covid pandemic) can maximize their impact<br>• Replies, mentions, and other techniques that target individuals, were found to consistently and largely reduce retransmission and should be used only if they are actually necessary	0.89

Slavik *et al.*	2021	CA	Define tweeting practices during public health crises to improve risk communication and maximise engagement.	Twitter	n/a	Mixed Method	Tweets (485)	6 months (1 January 2020 - 30 June 2020)	Engagement	Risk communication strategies were not widely used in tweets by any account type. Tweets using at least one risk communication strategy (n=262) received an average of 61 retweets per tweet, while tweets using no risk communication strategies received an average of 13. Efficacy and risk statements were the most commonly used strategies.	Need for public health agencies and decision makers to monitor Twitter analytics in order to understand their audience and leverage whatever Twitter engagement strategies help maximize shares of their communications.	0.70
Sutton *et al.*	2020	US	Identify and describe the patterns of longitudinal risk communication from public health communicating agencies on Twitter during the first 60 days of the response to the novel coronavirus disease 2019 (COVID-19) pandemic.	Twitter	n/a	Content Analysis	Tweets (138,546)	2 months (1 February 2020 - 31 March 2020)	Message framing	Hashtags, grouped according to the time period in which they emerged and persisted, provided a 7 periods of communication emphases: lead up, acute onset, individually focused action, community focused action, sustaining motivation, setting social norm, secondary impacts.	• Public health-oriented hashtag campaigns may help engage individuals to help them to feel part of a larger collective body and to participate locally by contributing information about their local context<br>• Planning for rapid coordination, use of common language, and targeted boosting of critical communications is important for successful response to events	0.79
Sutton *et al.*	2020	US	Identify strategies and practices that may lead to increased message passing among online users.	Twitter	n/a	Content Analysis	Tweets (149,335)	3 months (1 February 2020 - 30 April 2020)	Engagement	• Type of organization posting messages, timing of the message, follower numbers, has effects on message retransmission<br>• The inclusion of hashtags increase message passing<br>• The inclusion of media, videos and photos, lead to significantly greater message sharing<br>• Message with content about individual efficacy were passed on more often. Information sharing, the use of exclamation mark, the inclusion of a URL, quote tweets decrease retransmission<br>• The inclusion of directed messages, in the form of replies, consistently and negatively affects message retransmission under conditions of imminent threat<br>• Tweets containing content about surveillance, referencing the extent of those infected, hospitalized, dead, or recovered, and tweets containing technical information, both positively affect message retransmission	In a uncertain environment, public agecies can reach the public - and increase message sharing - with a wide range of practical information regarding the health impacts of COVID19, protective action measures, and the progress of the pandemic itself. At the same time, some tactics useful in other disasters (such as sentence styles that use exclamatory and interrogative punctuation) are counter productive in the COVID-19 pandemic.	0.89
Tang et al.	2021	US	Examine the content of COVID-19–related tweets posted by public health agencies in Texas and how content characteristics can predict the level of public engagement.	Twitter	Health Belief Model (HBM)	Content analysis	Tweets (7,269)	n/a	Engagement	The information function was the most prominent function, followed by the action or community functions. Beliefs regarding susceptibility, severity, and benefits were the most frequently covered health beliefs. Tweets that served the information or action functions were more likely to be retweeted, while tweets that served the action and community functions were more likely to be liked. Tweets that provided susceptibility information resulted in the most public engagement in terms of the number of retweets and likes.	Public health agencies should continue to use Twitter to disseminate information, promote action, and build communities. They need to improve their strategies for designing social media messages about the benefits of disease prevention behaviors and audiences'self-efficacy.	0.72
Vos *et al.*	2018	US	Study the relationship between message passing and message content related to threat and efficacy and the theoretical predictors of risk message passing.	Twitter	Extended Parallel Process Model	Content Analysis	Tweets (1,409)	1 month (1 August 2016 - 30 August 2016)	Engagement	• Message content related to the severity of a threat and efficacy has the potential to increase the sharing of risk communication messages on Twitter<br>• Including both types of content in the same message creates a subadditive effect<br>• Including information about official action, using a keyword hashtag related to the event, and including an image increased message passing<br>• Saliency - as measured by the timing of the message - influences message passing	Public health officials may want to emphasize the severity of an emerging infectious disease. Efficacy information is an important message element in encouraging an effective response.	0.81

Vos *et al.*	2020	US	Analyse the emergency risk communication conceptual model in order to propose a method for evaluating emergency risk communication on social media.	Twitter	The emergency risk communication model developed by CDC	Content Analysis	Tweets (2,915)	28 days (1 October 2014 - 29 October 2014)	Engagement	• Scientifically accurate messages (operationalized as containing threat information), open and transparent messages (operationalized as containing official action anduse of the first person), and clear messages (operationalized as containing images and use of figurative language) influence audience behaviors<br>• The day the message was sent influenced message passing<br>• Messages sent by accounts with larger follower numbers exhibitedincreased retweeting	Organizations should create messages that illustrate information visually and should try to include threat and efficacy information in messages; they should also engage social media audiences before public health crises emerge.	0.84
Yang *et al.*	2021	CN	Indicate which factors in Wuhan Release's posts influenced citizen engagement on Weibo, in relation to content type, text length, infor-mation source, media richness, the dialogic loop of the text.	Sina Weibo	Media richness<br>Dialogic loop	Mixed method	Posts (3,596)	4 months (31 December - 19 April 2020)	Engagement	• Various types of messages play a different important role in every stage of the crisis<br>• The dialogic loop is partially associated with citizen participation<br>• The addition of pictures and links to information has a significant positive effect<br>• The scale of content is significantly positively correlated with citizen participation	• Authorities should release information depending on the varying needs of the public and the actual situation of the crisis • Government agencies should selectively employ media richness theory and actively use the dialogic loop to enhance public engagement<br>• Government accounts can take full advantage of social media functions, especially mentions and hashtags, the number of original posts, add pictures and text length appropriately, to increase interactions with the public, to improve the level of engagement	0.67
Young *et al.*	2018	US	Explore the operationalization of social media engagement during global health crises.	Twitter	Dialogic Communication	Content analysis	Public comments (295) Questions (385)	n/a	Engagement	• Chats were effective at answering questions about disease, creating a forum for targeted criticism, and promoting conversation among participants<br>• Chats as a method of engagement was not effective at promoting dialogic communication between the organization and its publics	• The question–response dyad is a form of ideal communication that suggests engagement but maintains organizational expertise<br>• Creating spaces for regular engagement online when health organizations are not handling outbreaks, could help for the informational engagement during crises	0.70

Table S2. List of studies on disease prevention and health promotion

References	Year	Country	Topic	Aim	Communication channel	Theories or models used (if any)	Study design	Sample	Observation time	Primary evaluated aspect	Main results	Proposed Strategy / Practical Implication	KMET
Alkazemi *et al.*	2020	BH, KW, OM, SA, AE	Health promotion	Analyse how public health information is discussed differently across the GCC on Instagram, as well as the extent to which Health Belief Model (HBM) varies. Also to what degree do Instagram users engage – in the form of "likes" and "comments" – with Instagram posts by the GCC states	Instagram	Health Belief Model (HBM)	Content analysis	Posts (1,000)	6 months - 2 years	Engagement	59% of the posts sample of posts were public relations related. The results of this study demonstrate that only benefits and cues to action were mentioned in more than 20% of the Instagram posts. All other constructs of the HBM were only present in approximately 6% of posts.	Include more HBM constructs. Develop a separate PR profile for HOs.	0.81
Allen *et al.*	2020	US	Disease prevention	Feasibility and acceptability of implementing a Twitter campaign to promote knowledge about the human papillomavirus (HPV) vaccine.	Twitter	Health Belief Model (HBM)	Observational	Low income women (35)	1 month	Campaign effectiveness	No statistically significant change in the intent to be vaccinated in the next 6-months ($p = 1.000$) or 12-months ($p = 0.617$) after the campaign among those who had not yet started or completed vaccination.	Women received a daily tweet over a period of one-month that contained messages from educational materials. Messages were selected to align with the Health Belief Model and primarily addressed Vaccination Content.	0.65
Alonso-Cañadas *et al.*	2020	ES	Health promotion	Improve HOs decision-making in communication strategies via social media, in order to achieve a greater social commitment.	Facebook	Media Richness Theory (MRT)	Content analysis	Posts (19,817)	1 year (1st January 2018 - 31st December 2018)	Engagement	The video format generates greater user participation, while making publications in other less interactive formats (link, note, music or status) has the opposite effect.	The message format affects the level of users' online commitment with HOs via social media.	0.84
Barklamb *et al.*	2020	AU	Health promotion	Identify the SM post strategies associated with higher engagement in nutrition and food-related posts.	Facebook Instagram	Social Marketing Techniques	Content analysis	Posts (736)	46 days (19 February - 5 April 2018)	Engagement	• FB: strategies that were associated with higher engagement included the use of hashtags and announcements compared to not prompting engagement strategies<br>• IN: not inducing any emotion/hedonic sensations, and using a negative tone had a negative impact on Instagram engagement	FB: Start using hashtags on posts, continue prompting engagement with users, specifically through 'announcements' when appropriate<br>IN: Consider engaging ways to encourage consumption of core foods or exercising, i.e., positive emotions, tone, and hedonic sensations	0.92
Borah *et al.*	2018	US	Health promotion	Understand the effect of health message framing and the moderating effects of social endorsement and source type on credibility perceptions of Facebook posts.	Facebook	Bandwagon heuristic	Experimental	College students (892)	n/a	Message framing	• A gain-framed message will be perceived as more credible than a loss-framed message<br>• Expert sources will be considered more credible<br>• Participants considered the gain-framed post by an expert source with the high number of 'likes' as the most credible message	Use of gain-framed messages	0.82

Cho *et al.*	2018	US	Health promotion	Define might be the critical elements in health promotion messages to encourage people to exercise or attend an event that promotes physical activities.	Website	Prospect Theory (PT)<br>Construal Level Theory (CLT)<br>Health Belief Model (HBM)	Experimental	Adults (138)	n/a	Message framing	• Participants who read a gain-framed message had higher campaign attitude and behavioural intention than those who read a loss-framed message<br>• Participants under societal appeal exhibitited a more positive attitude toward the campaign than those under individual appeal<br>• People who read a loss-framed message perceived higher severity when they saw an individual appeal, perceived severity was correlated with behavioural intention	• A gain-framed message appear to be more effective in messages meant to highlight benefits of a behaviour or promoting an event for physical exercise<br>• A loss-framed message is effective when it is paired with an individual appeal where perceived severity is highlighted	0.83
Chung	2017	US	Disease prevention	Define the factors that contribute to the wide dissemination of campaign messages on Twitter.	Twitter	n/a	Content Analysis	Tweets (1018)	1 month and 10 days	Engagement	• The use of photos or images increased the probability to be retweeted<br>• The number of followers, and the frequency of tweets were positively related to the frequency of retweets	• Frequent posting is beneficial<br>• For health promotion messages, using photos and images (such as infographics) instead of videos or hyperlinks may be beneficial	0.78
Chung *et al*.	2020	US	Disease prevention	• Examine whether dialogic messages induce greater risk-preventive behavioural intentions than monologic messages.<br>• Test if the effectiveness of dialogic (vs. monologic) messages, if any, varies across the message platform<br>• Examine the role of social presence and psychological distance to the health risk in forming risk-preventive behavioural intentions	Twitter<br>Website	Construal Level Theory (CLT)	Experimental	Twitter users (209)	n/a	Message framing	• In a Twitter page, dialogic and monologic risk messages did not exert significantly differential effects on perceived social presence. A dialogic message did generate a stronger social presence in a website page<br>• Participants who read dialogic messages indicated a significantly higher level of social presence than those who read monologic messages. Social presence was a significant predictor of psychological distance to the risk, a closer psychological distance to the risk was a significant predictor of risk-preventive behavioural intentions	• A website page is better suited for dialogic communication, a twitter post does greatly benefit from dialogic communication<br>• Dialogic messages, even in a primitive format like the Q&A section, may have a powerful impact on behavioural outcomes	0.82
Dockter *et al.*	2020	US	Health promotion	Investigate how retransmission (i.e., social media content shared by a familiar, credible organization) and modality (i.e., how the message is delivered) influence the effectiveness of communicating health research findings via social media.	Facebook	Elaboration Likelihood Model (ELM)<br>Dual Coding Theory (DCT)<br>MAIN model	Experimental	Adults (517)	n/a	Message framing	• Retransmitted health research communication results in higher perceived source credibility and perceived message effectiveness<br>• Health research communication retransmitted by a well-known, credible source will result in stronger intentions to engage with the Facebook content<br>• When retransmitted, the video modality resulted in significantly higher perceived credibility and perceived effectiveness, compared to the infographic modality	• Content retransmitted by other well-known, credible sources increases in credibility, message effectiveness, and engagement intentions.<br>• It may be useful to invest in the creation of short videos to communicate findings	0.81
Frisch *et al.*	2013	CH	Disease prevention	Investigate how retention of information differs for text-only presentation, image-only presentation, and multimedia (text and image) presentation of online health information	Website	Dual Coding Theory (DCT)	Experimental	University Students (240)	n/a	Campaign effectiveness	Students who were presented with the multimedia website version scored significantly higher on information retention than students presented with the text-only website version. No significant differences were found between the multimedia website version and the image only version.	Websites designed for health education should include visual presentations of information, such as pictures, charts, or graphs.	0.78
Guidry *et al.*	2020	US, AU, IN, CA, BR, CL, DE, IT, NG, ZA, SG, NZ	Disease prevention	• Define to what extent HBM constructs are used in national health department tweets across different nations, and how HBM constructs impact Twitter engagement<br>• Define to what extent visuals are used in national health department tweets across different nations, and how the visuals impact Twitter engagement	Twitter	Health Belief Model (HBM)	Observational	Tweets (1,200)	n/a	Engagement	• Health Belief Model (HBM) constructs were used frequently and, in most cases, increased public engagement in a statistically significant manner<br>• The use of visuals on Twitter tends to be associated with higher engagement	• Health communication professionals should use and adopt theoretical constructs when defining social media strategies and creating tweets in order to make these messages more effective towards digital publics.<br>• Health communication professionals should also utilize visuals such as images, videos, or infographics in their public health tweets in order	0.75

											to increase message engagement among citizens with low literacy.	
Harris *et al.*	2011	UK	Disease prevention	• Assess the factorial structure of a general measure of Web trust<br>• Model how the resultant factors predicted trust in, and readiness to act on, the advice found on health-related websites<br>• Test whether adding variables from social cognition models to capture elements of the response to threatening, online health risk information enhanced the prediction of these outcomes	Website	Extended Parallel Process Model (EPPM)<br>3-factor Model<br>Protection Motivation Theory (PMT)	Observational	Adults (561)	n/a	Campaign effectiveness	• Impartiality and information quality, had direct effects on trust in health-related websites<br>• Both threat and corroboration against other sources contributed to the prediction of trust	Four key design factors (information quality, personalization, perceived impartiality, and credible design) have an important (albeit moderated) role to play in influencing trust and the subsequent decision to act on the advice given.	0.70
Kim *et al.*	2016	US	Disease prevention	Investigate how different types of network structures explain retweeting behavior and which types of tweets are retweeted and why some messages generate more interaction among users.	Twitter	n/a	Content analysis	Tweets (7,000)	1 composite week (August 2011 - September 2011)	Engagement	The number of account followers, betweenness centrality, close- ness centrality, positive emotion, and tentativeness were all significantly associated with retweeting activity.	• Influential people shold be identified and targeted because their messages are more likely to be disseminated.<br>• Develop reliable messages about diagnosis, symptoms, and treatments with emotionally positive tones in an effort to capture the intended audiences' attention, associate an emotional state with coping behaviors, and promote social support and help people with health problems cope better	0.81
Kite *et al.*	2019	AU	Health promotion	Investigate usage of and engagement with the Make Healthy Normal Facebook page and to identify influential factors in driving engagement with the page.	Facebook	n/a	Content analysis	Posts (392)	2 years 3 months (June 2015 - September 2017)	Engagement	• Paying for posts significantly increases reach of posts, except for video content<br>• Organic impressions predicted higher engagement with the MHN page compared to paid impressions	Effective engagement through Facebook requires both maximizing the reach of posts through paid boosts and delivering content that users want to engage with and share in order to capitalize on word-of-mouth marketing.	0.78
Klassen *et al.*	2018	AU	Health promotion	Identify and quantify social media strategies used by the food industry and lifestyle brands, and health promotion organizations across the social networking sites Facebook and Instagram	Facebook<br>Instagram	n/a	Content analysis	Facebook posts (143)<br>Instagram posts (84)	30 days (August 2017 - September 2017)	Engagement	• Health promotion organizations frequently provided statistics and facts and used a more serious tone, while lifestyle brands were positive and relatable<br>• Positive emotion-inducing strategies were associated with more interactions on Facebook and Instagram and using an optimistic tone was associated with more interactions on Facebook<br>• Posts classified as "relatable" were negatively associated with Facebook interactions but positively associated with Instagram interactions<br>• Videos and body image content were statistically significantly positively associated with Facebook interactions, but not associated with Instagram interactions	Health promotion organizations should try to build relationships with their users in a similar fashion as the lifestyle brands do.	0.89
Lister *et al.*	2015	US	Health promotion	Examine the use of low-cost social media platforms in communicating public health messages and outline the Laugh Model.	Website<br>Facebook<br>Twitter	The Laugh Model	Observational	"Family Meal Month" campaign	1 month (September 2014)	Engagement	The campaign was extremely cost-effective. The cost per person reached was 0.2 cents, with a cost of 0.3 cents per post impression. The overall cost per engagement was $0.27, with likes and follows costing $1.14 each and each post engagement costing 0.36 cents.	• Empowering and engaging techniques may be more effective than use of educational techniques<br>• Social media are often preferable to mass media in terms of cost-effectiveness, assessments of campaign success, and increased sustainability	0.60

Loft	2020	DK	Disease prevention	Assess how different Facebook posts resonate with parents hesitant about HPV vaccination and how to engage parents in positive dialogues on FB.	Facebook	Heart-brain communication	Content analysis<br>Focus group interviews	Posts (84)	7 months (May 2017 - December 2017)	Engagement	• Higher reach and more positive sentiments were achieved by posts that contained personal stories compared to those containing factual information<br>• Focus groups where the participants requested more in-depth information, including the safety of the vaccine<br>• In the focus groups parents hesitant of vaccination read comments and replies to posts on FB, even when they did not interact with the posts themselves	• Designing factual posts so that they include an emotional dimension increase the engagement with these posts<br>• Sponsored content can generate more negative comments than organic content<br>• All people should be addressed in an accommodating manner, regardless of their tone of voice	0.71
Nguyen *et al.*	2017	NL	Disease prevention	investigate the effect of a website tailored to individual preferences for the mode of information presentation, compared to 4 non-tailored websites on younger and older adults' attention and recall of information.	Website	Cognitive Theory of Multimedia Learning (CTML)	Experimental	Adults (559)	n/a	Campaign effectiveness	Mode tailoring enhanced older adults' recall of information via increased attention to the web-site, whereas younger adults recall slightly more information from a fixed website with text only or text with illustrations.	Mode tailoring may be a tool to reduce or prevent the information overload that may occur when too much information (via text, illustrations, and video) is placed on a non- tailored webpage at one time, and facilitate information processing for older adults.	0.89
Nobles *et al.*	2020	US	Disease prevention	Apply automated image recognition software to examine the demographic profile of faces in Instagram posts containing the hashtag #HIV and compare this to the demographic break- down of those most at risk of a new HIV diagnosis.	Instagram	n/a	Content analysis	Posts (10,036)	18 months (January 2017 - July 2018)	Message framing	Faces skewed older (47% vs. 11% were 35–39 years old), more female (41% vs. 19%), more white (43% vs. 26%), less black (31% vs 44%), and less Hispanic (13% vs 25%) on Instagram than for new HIV diagnoses.	HIV prevention and control professionals could introduce more demographically representative images by purchasing sponsored Instagram posts or developing public-facing accounts.	0.75
Parackal *et al.*	2017	NZ	Disease prevention	• Examine user engagement for a public health campaign based on the metadata provided by Facebook<br>• Analyse comments generated by the campaign materials using text mining<br>• Investigate the relationship between the themes identified in the comments and the message and the sentiments prevalent in the themes that exhibited significant relationships	Facebook	The Laugh Model	Thematic Analysis	Comments (819)	3 months (June 2015 - September 2015)	Engagement	• The overall number of likes (6125), shares (300), and comments (819) achieved in a short time (June to September 2015) suggests the promotional material stimulated reasonable engagement within the target audience<br>• Comments were mostly negative (66%)	• Communication effectiveness could be enhanced using a two-way communication format, which enables the promoter to respond to the negative comments<br>• Public campaigns on social media should include a protocol against moderating the comments solely based on sentiment valence so as to maximize the intervening opportunities and maintain the completeness and quality of the raw data	0.75
Parackal *et al.*	2020	NZ	Disease prevention	Investigate the suitability of a dynamic transactional model (DTM) of communication for explaining the use of social media for communicating public health messages.	Facebook	Dynamic Transactional Model (DTM)	Thematic Analysis	Comments (819)	3 months (June 2015 - September 2015)	Message framing	• The plurality of topics in the comments was verified, which aligns with the DTM assumption that parties in communication simultaneously send and receive information (inter-transaction)<br>• The heterogeneity of sentiment observed for the three topics confirmed a polarity of sentiment even at the topic level	The observations reported in this study suggest a DTM could serve as a framework for implementing health promotion via Facebook.	0.64
Perrault *et al.*	2014	US	Disease prevention	Attempt to determine the effectiveness of adding four short videos to a website promoting the reduction of PFOA in people's homes.	Website	Social Cognitive Theory (SCT)<br>Media Richness Theory<br>Cue Summation Theory	Experimental	Mothers (197)	n/a	Campaign effectiveness	• Individuals exposed to the video condition reported engaging in one additional risk reduction behavior than the control condition<br>• Those in the video condition believed they could more effectively and easily perform the recommended behaviors than those in the non-video condition	For practitioners, to maximize their investment, time and energy, it would probably be best to spend on making one really effective video to place on a homepage	0.69
Pirzadeh *et al.*	2020	IR	Health promotion	Examine the effects of a web-based intervention on the promotion of physical activity among adolescents using the Trans Theoretical Model.	Website	Trans Theoretical Model (TTM)	Experimental	High school students (278)	n/a	Campaign effectiveness	Participants showed significant progress during stages of change, and changes were greater in the group who was trained by TTM.	Website based education on PA can be effective. Education based on the TTM that applies constructs such as self-efficacy, will be more useful on behavior change.	0.68

Potente	2011	AU	Disease prevention	Determine whether entertainment-education strategies could be used in a communication campaign to engage youth, encouraging peer-to-peer dialogue about skin cancer prevention and improving attitudes to effective sun protection.	YouTube MySpace Facebook	Entertainment-education Social media marketing	Observational	Partecipants exposed to the video (667) Partecipants not exposed (961)	4 Months (December 2008 - March 2009)	Campaign effectiveness	Of those who watched the video, 25% forwarded the video to friends, 44% reported that the video changed the way they felt about sun protection, and 19% of spontaneous online comments reported an intention to change behavior.	Social media can be used effectively in social marketing campaigns and is an essential tool in the promotional mix when targeting young people. Entertaining, peer-to-peer messages can be used to engage youth with an important health message for skin cancer prevention	0.78
Rabarison *et al.*	2017	US	Health promotion	• Analyse whether Twitter chats facilitate engagement in two-way communications between public health entities and their audience<br>• Describe how to measure two-way communications, incoming and outgoing mentions, between users in a protocol using free and publicly available tools (Symplur, OpenRefine, and Gephi)	Twitter	n/a	Content analysis	Tweets (744)	n/a	Engagement	• #LiveFitNOLA was the sixth trending hashtag in New Orleans on March 5th, 2015<br>• @TulanePRC was the main driver of this chat and had a 40% ROE	• Public health entities can use Twitter chats as a two-way communication audience engagement tool with their audience<br>• In future chats, the chat organizers should encourage guest hosts to share original content and actively respond to the chat participants	0.75
Reuter *et al.*	2021	US	Disease prevention	Assess the difference in audience engagement with identical antismoking health messages on three social media sites (Twitter, Facebook, and Instagram) and with a referring link to a tobacco prevention website cited in these messages.	Twitter Facebook Instagram	Selective Exposure Self- and Affect-Management (SESAM) affordance theory	Content analysis	Posts (1275)	84 days (19 April 2020 - 12July 2017)	Engagement	• The click-through rate (CTR)for Instagram was the highest, followed by Facebook and Twitter<br>• Twitter had the highest wCTRs (website page visit including scrolling to peruse content), followed by Facebook<br>• Organic messages had a higher CTR but lower wCTR than paid advertisements	• Engagement with a health message on social media (CTR) does not indicate user engagement on the website (wCTR), it is recommended that both metrics should be taken into account when designing health promotion strategies<br>• Combining organic and advertising messages in health promotion campaigns could be advantageous	0.75
Rus *et al.*	2016	US	Disease prevention	Conduct a content analysis of health communications posted on diabetes-related Facebook pages and explore the role of crowdsourcing (direct solicitation of user input) and use of external links and videos.	Facebook	Common-Sense Model of Illness Self- Regulation	Content analysis	Posts (500)	3-49 days	Engagement	• Imagery was the strongest predictor of both liking a health communication and sharing it with others but it also reduced the number of comments when paired with causal information • The use ofimagery attenuated or reversed the positive relationships of diabetes consequence information, control information, nega- tive affect, positive identity, and social support with user engagement<br>• Messages with expressions of negative affect were shared less frequently relative to mes- sages without negative affect<br>• External links were associated with lower engagement	•Imagery should be carefully employed since it appears to be a powerful tool for attracting attention and briefly engaging users (i.e., in- creasing likes) as well as increasing message transmission (i.e., increasing shares).However, use of images with information about illness consequences and control or with messages conveying negative affect could mute responses.	0.84
Strekalova *et al.*	2017	US	Disease prevention	Evaluate what strategies are most effective for engaging audiences.	Facebook	Information Richness Theory (IRT)	Content Analysis	Posts and their associated data (1,975) comments (4,537)shares (77,298) likes (145,462)	4 years and 7 months (July 2010 - February 2015)	Engagement	Audience engagement is associated with the format of cancer-related social media post. Posts with photos received significantly more likes, comments, and shares than videos, links, and status updates.	Photos with short comments are the most effective in engaging information consumers, and greater use of this post type could encourage greater audience engagement. At the same time, professional videos may not be as effective as a mechanism for active audience engagement on social media platform.	0.78
Syred	2014	UK	Health promotion	Examine which elements of moderator and participant behavior stimulated and maintained interaction with a sexual health promotion site on Facebook.	Facebook	n/a	Content Analysis	Participants (576)	5 months (18 January 2010 - 27 June 2010)	Engagement	The moderator choose from a list of standardized responses; there was no attempt to engage in the conversation or stimulate discussion; allowed time for other posters to correct misinformation and then endorsed the corrections. User-generated content did not resolve uncertainty around some aspects of chlamydia testing: this maintained conversations by generating disagreement and encouraged sharing personal experience.	Moderation for online health promotion interventions: (1) unengaged and professional oversight to provide a safe space for discussion and to maintain information quality and (2) a more engaged and interactive presence designed to maintain interest that generates new material for discussion and is responsive to user requests.	0.64
Theiss	2016	UK	Disease prevention	Describe the impact of social media efforts on health communication.	Facebook	n/a	Content Analysis	Posts (574)	20 months (August 2014 - mid-April 2016)	Engagement	Engagement rate was highest for content shared for the Know: BRCA campaign posts, followed by the non-campaign related posts, and the Bring Your Brave posts. Overall engagement rate decreased from 2014–2016. Photos consistently produced the most significant engagement rate overall.	Users were more likely to click, share, comment, or like the content of the post that had photos. Branded, visual content is more effective in facilitating engagement.	0.84

Whitten *et al.*	2011	US	Disease prevention	Address the presence of information targeting low literacy, racially diverse, non-English speaking, and age diverse audiences on breast cancer websites. Also to document the utilization of evidence strategies, either statistics or storytelling, for these audiences.	Website	Targeted communication, Statistical and story telling evidence	Content Analysis	Breast cancer websites (157)	n/a	Message framing	• Nearly half of websites did not contain any diversity elements<br>• Language diversity, was found on only 30% of the websites<br>• Websites using story telling evidence to convey breast cancer messages were vastly out numbered by those using statistical evidence	• If content were tagged according to ethnicity or language, then this would allow users to browse websites according to the information that is most personally relevant<br>• It would be beneficial for websites containing lower literacy material and to avoid statistics<br>• Story telling evidence has demonstrated the ability to serve as a greater motivator for healthy behaviors	0.56
Yoo *et al.*	2020	KR	Disease prevention	• Explicate the cognitive mechanism underlying the process through which exposure to risk information on social media shapes people's behavioral intentions<br>• Determine whether exposures to risk information on two different types of social media (content-oriented social media focusing on shared interests vs user-oriented socialmedia focusing on social relationship) have different effects on people's risk perceptions and behavioral intentions	Twitter<br>YouTube<br>Instagram<br>Flickr<br>Facebook<br>KakaoStory<br>Naver Band	Uses and Gratifications Theory<br>Social<br>Media Ecology Model	Observational	Participants (688)	11 days<br>(8 December 2016 - 19 December 2016)	Message framing	• Content-oriented social media exposure was related to both personal- and societal-level risk perceptions.<br>• Only content-oriented social media exposure led — indirectly via risk perception — to behavioral intentions<br>• User-oriented social media exposure had no impact on risk perception and behavioral intentions	Content-oriented social media may be more effective in influencing risk perceptions and should be employed for risk messages and campaigns that aim to shape risk perceptions.	0.78
Yoo *et al.*	2018	US	Disease prevention	• Develop a health communication campaign model for social media and test itsutility<br>• Investigate how social media channel-related per-ceptions affect preventive health behaviors • Investigate how communicative behavior mediates health-related and channel-specific perceptions and behavioral intention	Twitter	Situational Theory of Problem Solving (STOP)<br>Theory of Planned Behavior (TPB)	Observational	Females (220)	n/a	Campaign effectiveness	• Perceived risk and self-efficacy have direct, rather than indirect, impacts on beha-vioral intention<br>• The information acquisition mediated perceived risk's effect on intention and mediated the relationships between intention and information selection and transmission<br>• Social media-related perceptions also had indirect impacts on behavioral intention through communicative behaviors	• Need to consider incorporating features such as hyperlinks to related websites or live chat with health care providers<br>• Tailored messages or edutainment may also need to be considered to engage people in the process of information selection and transmission	0.70
Zhang	2019	US	Disease prevention	Assessing direct causal insight into the most effective strategies for designing messages over an online social media platform about cervical cancer prevention.	Twitter<br>Anonymous online discussion platform	n/a	Observational	Tweets from the observational study (462)<br>Created additional set (438)	4 years and 5 days<br>(1 January 2010 - 31 December 2014 + 5days)	Engagement	• Personal experience tweets were associated with more retweets in comparison with factual in-formation tweets. tweets from individual senders wereassociated with fewer retweets in comparison to those from organiza-tional senders<br>• Tweets with factual information and from organizational senders were significantly more likely to produce shares. Each of these factors was found to have an independent causal effect onmessage sharing behavior	In the social media contexts, the relative importance of having clear informational content sent from organizations will be much greater for generating highly viewed and highly shared cancer prevention messages	0.93
Zhang *et al.*	2020	US	Disease prevention	Examine content variables, including threat and efficacy, and user variables, including processing style and image promotional consideration, to investigate the combined influence in driving SNS users' intention to share fear appeal messages.	Facebook	n/a	Experimental	Studentsof Southeast Public University (214)	25 days<br>(11 January 2017 - 5 February 2017)	Engagement	• A high threat message elicits higher intention to share on social media as compared to a low threat message<br>• Fear is positively related to users' intention to share on social media<br>• Willingness to create a positive image of oneself on Facebook positively predicts intention to share the fear appeal message	• Consider including more emotional cues, such as pictures, to arouse fear in order to motivate information dissemination on SNS<br>• Try to reduce sensitive content that might hurt image if the aim is to get the risk message shared publicly	0.86

References	Year	Country	Aim	Communication channel	Theories or models used (if any)	Study design	Sample	Observation time	Primary evaluated aspect	Main results	Proposed Strategy / Practical Implication	KMET
Bhattacharya	2014	US	Investigate factors associated with engagement of US Federal Health Agencies via Twitter.	Twitter	n/a	Observational	Federal Health agencies (25)	Variable<br>3 months - 3 years	Engagement	Hashtags, URLs and user-mentions are positively associated with retweets.	Use of post containing hashtags, URLs and user-mentions.	0.84
Bhattacharya *et al.*	2017	US	Identify factors associated with Facebook engagement.	Facebook	n/a	Observational	Posts (45,852)<br>Federal Health agencies (24)	4 years<br>(January 2013 - 2017)	Engagement	Posts containing media or links and expressing positive sentiment correlate with higher or longer engagement.	Use of post containing media or links and expressing positive sentiment.	0.70
Campbell *et al.*	2020	UK	Determine factors that could affect the uptake of global health topics on social media.	Facebook	n/a	Observational	Videos (10)	2 months	Engagement	Three of the videos managed to raise sufficient attention to be shared further by a newspaper online portal. One video (the "dramatic" one) went viral. Viewers need to find the content particularly interesting or useful to them personally. Enhancing factors: well-known presenter, a "doomsday" topic, the presence of a controversy and an ongoing public debate.	Adjust the language and presentation used to be of more appeal to popular culture. Linking promotional materials to other popular topics could prove to be a successful strategy to achieve this.	0.40
Fung *et al.*	2018	US	Describe the contents and followers of the Twitter profile @CDC_AMD and to assess if attaching photos or videos to tweets posted by @CDC_AMD would increase retweet frequency.	Twitter	n/a	Observational	Tweets (768)	18 months<br>(5 May 2015 - 16 November 2016)	Engagement	Attaching photos or videos to original @CDC_AMD tweets increases the number of retweets by 37%. Content topics did not explain or modify this association.	Attaching visual cues to tweets will increase their retweet frequency, and this practice may enhance Twitter users' engagement with health communication messages promoted by medical and public health professionals.	0.75
Hung *et al.*	2011	CN, TW, UK, US	• Determine the critical categories for a content analysis<br>• Develop evaluation guidelines for a children's health website<br>• Compare the differences between children's eHealth design in the East and the West	Website	n/a	Content analysis	Websites (40)	n/a	Message framing	• In terms of information design, credibility is better presented in the West than in the East, whereas users' needs are better presented in the East than in the West.<br>• In terms of multimedia design, demonstrations and humanity are better presented in the West than in the East, and the segmentation of demonstrations is valued by both the East and the West<br>• In terms of interface design, the West presents a better appearance than the East, and methodology is valued by both the East and the West	• General terms should replace the professional term<br>• Simplified text-based content should be used<br>• Advertising space, wich may increase users' cognition load and mislead patients' treatment decisions, must be reduced<br>• Hi-Tec multimedia design, which may decrease the amount of explanatory text and increase the cognitive load, must be reduced	0.63

Kim *et al.*	2020	US	Explore the use of Instagram by the Centers for Disease Control and Prevention.	Instagram	n/a	Content analysis	Posts (1,545)	n/a	Engagement	• The most frequent type of photo was text mainly about calls for actions, detailed information were usually listed in post texts<br>•The mean engagement and the mean comment sentiments of text categorized photos were not relatively high<br>• Photos with more and bigger human faces had lower level of engagement and an higher level of comment sentiment, happiness and neutral expressed on the faces in photos were negatively associated with engagement.<br>• The variances of RGB, the variances of saturation and value, share of warm colors, contrast, and RGB contrast, were negatively correlated with engagement	The message design should be different depending on whether they aim to increase the number of likes and comments or they aim to induce more positive response from public.	0.73
Kite *et al.*	2016	AU	Identify the features of Facebook posts that are associated with higher user engagement on Australian public health organisations' Facebook pages.	Facebook	n/a	Content analysis	Posts (5,356)	1 year<br>(6 September 2014 - 31 August 2015)	Engagement	• Video posts were the most engaging post type, shared on average four times more often than photo posts<br>• Links and text-only posts received fewer likes and shares than photo posts<br>• Per impression, a photo attracted greater engagement than a video post<br>• Sponsorships and partnerships and use of persons of authority resulted in fewer likes, shares, and comments, compared to posts with no marketing elements at all	Content providers must encourage engagement and adapt to the Facebook algorithm in order to maximise message exposure, while also ensuring that the content is of high quality.	0.86
Lazard *et al.*	2014	US	Highlight the influential role of design complexity for users' first impressions of health websites.	Website	Visual Complexity Theory (VCT)<br>Technology Acceptance Model (TAM)	Experimental	University students (231)	n/a	Message framing	High design complexity was associated to significantly greater perceptions of design esthetics, a better attitude towards the website, a greater percieved comprehensibility, a greater percieved usefulness, a greater message quality and higher perceptions of visual informativeness.	Designers and developers should use the guiding principles of design complexity to increase the likelihood that the informational content is being perceived as pleasurable and valuable to targeted audiences	0.84
Meng *et al.*	2018	US	Examine the impacts of perceived message features and network characteristics on size and structural virality of information diffusion on Twitter.	Twitter	Diffusion of Innovations (DOI)<br>Information Utility Theory (IUT)	Content analysis	Tweets (425)<br>Retweeters (10,035)	17 weeks<br>(7 April 2015 - 4 August 2015)	Engagement	• The perceived efficacy and negative emotion as a message feature positively predict the number of retweets received by the message<br>• The perceived susceptibility, negative emotion as a message feature and the involvment of brokers significantly increase structural virality	• Designing efficacious information is the key to increase the aggregated number of retweets<br>• Crafting information that can raise risk perception is important to increase the diffusion chain through person-to-person transmission<br>• Tweets that induce negative emotions could be more effective in catching users' attention and expanding sharing of the information	0.84
Pang *et al.*	2016	AU	Create a design for consumer health websites by supporting different health information–seeking behaviours.	Website	n/a	Experimental	Adults (31)	n/a	Engagement	Partecipants used different search tactics for different search tasks, even when different tasks employed the same website.	• Access to a dynamic information scope is critical for health information seeking<br>• In many cases, casual seekers do not have a specific search target; hence, a fuzzy query approach can increase the chance of returning information that is interested to the user	0.86
Park *et al.*	2016	US	Examine how major health organizations use Twitter for disseminating health information, building relationships, and encouraging actions to improve health.	Twitter	Dialogic Theory (DT)	Content analysis	Tweets (1,583)	1 year<br>(September 2012 - August 2013)	Engagement	• Nearly 70% of the tweets appeared to be original posts, followed by retweets (18.0%) and replies (13.3%)<br>• Frequency of output is not antecedent to conservation of followers	• The number of retweets as a way to operationalize usefulness should be considered when applying dialogic theory to Twitter<br>• Organizations should consider retweeting content from health information sources with a high number of Twitter followers if they want to build up their own follower base	0.78

Shim *et al.*	2020	KR	Identify what quality factors matter in evaluating online health information sites which in turn affect users' benefits, applying the updated DeLone and McLean Model of Information Systems Success.	Website	Information Systems Success Model	Observational	Adults (506)	n/a	Message framing	Information quality had significant associations with all outcome variables: user satisfaction, intention to reuse the site, and perceived benefits of site use in health settings. Service quality had a significant association with user satisfaction, and its impact on perceived benefits occurred indirectly through user satisfaction and intention.	It is important to build trusted relationships with site usersand appropriately respond to their demands. Both the quality of the information provided on a health information site and the quality of service available on the site play significant roles in increasing users' perceived benefits.	0.89
Sillence *et al.*	2019	US, UK	Identify the key factors influencing US and UK citizens' trust and intention to act on advice found on health websites and to understand the role of patient experiences.	Website	n/a	Observational	Adults (1123)	n/a	Message framing	Trust was found to have a significant direct effect on intention to act, and of the trust factors, only credibility and impartiality had a significant direct effect on trust. The impact of personal experiences on trust was mediated through information corroboration.	Impartiality continues to remain a key predictor of trust in health websites as well as the extent to which users consider information sources to be credible. The presence of personal experiences information can have a positive influence on trust provided that users corroborate the information through additional sources.	0.81

Table S3. List of studies on general health

References	Year	Country	Aim	Communication channel	Theories or models used (if any)	Study design	Sample	Observation time	Primary evaluated aspect	Main results	Proposed Strategy / Practical Implication	KMET
Bhattacharya	2014	US	Investigate factors associated with engagement of US Federal Health Agencies via Twitter.	Twitter	n/a	Observational	Federal Health agencies (25)	Variable 3 months - 3 years	Engagement	Hashtags, URLs and user-mentions are positively associated with retweets.	Use of post containing hashtags, URLs and user-mentions.	0.84
Bhattacharya *et al.*	2017	US	Identify factors associated with Facebook engagement.	Facebook	n/a	Observational	Posts (45,852) Federal Health agencies (24)	4 years (January 2013 - 2017)	Engagement	Posts containing media or links and expressing positive sentiment correlate with higher or longer engagement.	Use of post containing media or links and expressing positive sentiment.	0.70
Campbell *et al.*	2020	UK	Determine factors that could affect the uptake of global health topics on social media.	Facebook	n/a	Observational	Videos (10)	2 months	Engagement	Three of the videos managed to raise sufficient attention to be shared further by a newspaper online portal. One video (the "dramatic" one) went viral. Viewers need to find the content particularly interesting or useful to them personally. Enhancing factors: well-known presenter, a “doomsday” topic, the presence of a controversy and an ongoing public debate.	Adjust the language and presentation used to be of more appeal to popular culture. Linking promotional materials to other popular topics could prove to be a successful strategy to achieve this.	0.40
Fung *et al.*	2018	US	Describe the contents and followers of the Twitter profile @CDC_AMD and to assess if attaching photos or videos to tweets posted by @CDC_AMD would increase retweet frequency.	Twitter	n/a	Observational	Tweets (768)	18 months (5 May 2015 - 16 November 2016)	Engagement	Attaching photos or videos to original @CDC_AMD tweets increases the number of retweets by 37%. Content topics did not explain or modify this association.	Attaching visual cues to tweets will increase their retweet frequency, and this practice may enhance Twitter users’ engagement with health communication messages promoted by medical and public health professionals.	0.75
Hung *et al.*	2011	CN, TW, UK, US	• Determine the critical categories for a content analysis<br>• Develop evaluation guidelines for a children’s health website<br>• Compare the differences between children’s eHealth design in the East and the West	Website	n/a	Content analysis	Websites (40)	n/a	Message framing	• In terms of information design, credibility is better presented in the West than in the East, whereas users' needs are better presented in the East than in the West. • In terms of multimedia design, demonstrations and humanity are better presented in the West than in the East, and the segmentation of demonstrations is valued by both the East and the West<br>• In terms of interface design, the West presents a better appearance than the East, and methodology is valued by both the East and the West	• General terms should replace the professional term • Simplified text-based content should be used<br>• Advertising space, wich may increase users’ cognition load and mislead patients’ treatment decisions, must be reduced<br>• Hi-Tec multimedia design, which may decrease the amount of explanatory text and increase the cognitive load, must be reduced	0.63

Kim *et al.*	2020	US	Explore the use of Instagram by the Centers for Disease Control and Prevention.	Instagram	n/a	Content analysis	Posts (1,545)	n/a	Engagement	• The most frequent type of photo was text mainly about calls for actions, detailed information were usually listed in post texts<br>•The mean engagement and the mean comment sentiments of text categorized photos were not relatively high<br>• Photos with more and bigger human faces had lower level of engagement and an higher level of comment sentiment, happiness and neutral expressed on the faces in photos were negatively associated with engagement.<br>• The variances of RGB, the variances of saturation and value, share of warm colors, contrast, and RGB contrast, were negatively correlated with engagement	The message design should be different depending on whether they aim to increase the number of likes and comments or they aim to induce more positive response from public.	0.73
Kite *et al.*	2016	AU	Identify the features of Facebook posts that are associated with higher user engagement on Australian public health organisations' Facebook pages.	Facebook	n/a	Content analysis	Posts (5,356)	1 year<br>(6 September 2014 - 31 August 2015)	Engagement	• Video posts were the most engaging post type, shared on average four times more often than photo posts<br>• Links and text-only posts received fewer likes and shares than photo posts<br>• Per impression, a photo attracted greater engagement than a video post<br>• Sponsorships and partnerships and use of persons of authority resulted in fewer likes, shares, and comments, compared to posts with no marketing elements at all	Content providers must encourage engagement and adapt to the Facebook algorithm in order to maximise message exposure, while also ensuring that the content is of high quality.	0.86
Lazard *et al.*	2014	US	Highlight the influential role of design complexity for users' first impressions of health websites.	Website	Visual Complexity Theory (VCT)<br>Technology Acceptance Model (TAM)	Experimental	University students (231)	n/a	Message framing	High design complexity was associated to significantly greater perceptions of design esthetics, a better attitude towards the website, a greater percieved comprehensibility, a greater percieved usefulness, a greater message quality and higher perceptions of visual informativeness.	Designers and developers should use the guiding principles of design complexity to increase the likelihood that the informational content is being perceived as pleasurable and valuable to targeted audiences	0.84
Meng *et al.*	2018	US	Examine the impacts of perceived message features and network characteristics on size and structural virality of information diffusion on Twitter.	Twitter	Diffusion of Innovations (DOI)<br>Information Utility Theory (IUT)	Content analisys	Tweets (425)<br>Retweeters (10,035)	17 weeks<br>(7 April 2015 - 4 August 2015)	Engagement	• The perceived efficacy and negative emotion as a message feature positively predict the number of retweets received by the message<br>• The perceived susceptibility, negative emotion as a message feature and the involvment of brokers significantly increase structural virality	• Designing efficacious information is the key to increase the aggregated number of retweets<br>• Crafting information that can raise risk perception is important to increase the diffusion chain through person-to-person transmission<br>• Tweets that induce negative emotions could be more effective in catching users' attention and expanding sharing of the information	0.84
Pang *et al.*	2016	AU	Create a design for consumer health websites by supporting different health information–seeking behaviours.	Website	n/a	Experimental	Adults (31)	n/a	Engagement	Partecipants used different search tactics for different search tasks, even when different tasks employed the same website.	• Access to a dynamic information scope is critical for health information seeking<br>• In many cases, casual seekers do not have a specific search target; hence, a fuzzy query approach can increase the chance of returning information that is interested to the user	0.86
Park *et al.*	2016	US	Examine how major health organizations use Twitter for disseminating health information, building relationships, and encouraging actions to improve health.	Twitter	Dialogic Theory (DT)	Content analysis	Tweets (1,583)	1 year<br>(September 2012 - August 2013)	Engagement	• Nearly 70% of the tweets appeared to be original posts, followed by retweets (18.0%) and replies (13.3%)<br>• Frequency of output is not antecedent to conservation of followers	• The number of retweets as a way to operationalize usefulness should be considered when applying dialogic theory to Twitter<br>• Organizations should consider retweeting content from health information sources with a high number of Twitter followers if they want to build up their own follower base	0.78

Shim *et al.*	2020	KR	Identify what quality factors matter in evaluating online health information sites which in turn affect users' benefits, applying the updated DeLone and McLean Model of Information Systems Success.	Website	Information Systems Success Model	Observational	Adults (506)	n/a	Message framing	Information quality had significant associations with all outcome variables: user satisfaction, intention to reuse the site, and perceived benefits of site use in health settings. Service quality had a significant association with user satisfaction, and its impact on perceived benefits occurred indirectly through user satisfaction and intention.	It is important to build trusted relationships with site usersand appropriately respond to their demands. Both the quality of the information provided on a health information site and the quality of service available on the site play significant roles in increasing users' perceived benefits.	0.89
Sillence *et al.*	2019	US, UK	Identify the key factors influencing US and UK citizens' trust and intention to act on advice found on health websites and to understand the role of patient experiences.	Website	n/a	Observational	Adults (1123)	n/a	Message framing	Trust was found to have a significant direct effect on intention to act, and of the trust factors, only credibility and impartiality had a significant direct effect on trust. The impact of personal experiences on trust was mediated through information corroboration.	Impartiality continues to remain a key predictor of trust in health websites as well as the extent to which users consider information sources to be credible. The presence of personal experiences information can have a positive influence on trust provided that users corroborate the information through additional sources.	0.81

Table S4. List of studies on misinformation correction and health literacy

References	Year	Country	Topic	Aim	Communication channel	Theories or models used (if any)	Study design	Sample	Observation time	Primary evaluated aspect	Main results	Proposed Strategy / Practical Implication	KMET
Bode *et al.*	2017	US	Misinformation correction	Investigate misinformation correction strategies.	Facebook (simulated news feed)	Conspiracy Beliefs	Experimental	University students (136)	n/a	Campaign effectiveness	Corrective information via an algorithm or social connections reduce misinformation and are effective and they are considered credible.	Correction via related stories algorithm or via social contacts.	0.66
Chin *et al.*	2018	US	Health literacy	Develop a systematic approach to improve web-based health information so that it is better understood and used by older adults with vary- ing health literacy abilities.	Website (on paper)	Process– Knowledge framework	Experimental	Older Adults (128)	n/a	Message framing	A multi-faceted approach to redesigning health information guided by theories of text comprehension and document design improves older adults' memory for self-care information typically found on credible websites.	Increasing document readability alone, is insufficient for improving older patients' comprehension.	0.81
Gesser-Edelsburg A.	2018	IL	Misinformation correction	• Examine ways for health organizations to correct misinformation concerning the measles vaccination on social networks for two groups: pro-vaccination and hesitant<br>• Examine the types of reactions of two subgroups (pro-vaccination, hesitant) to misinforma- tion correction<br>• Examine the effect of misinformation correction on these two sub- groups regarding reliability, satisfaction, self-efficacy and intentions	Facebook	Convergence Communication Approach	Experimental	Students (243)	n/a	Message framing	• The average reliability and satisfaction was significantly higher for subjects that had read the experimental condition correction post rather than the subject that had read the traditional correction post<br>• Even the pro-vaccination group prefers to receive full information that addresses emotions and concerns and expresses less satisfaction with a response that does not address those aspects	The findings indicate that it is very important for organizations to correct misinformation transparently while addressing the emotional aspect both for the pro-vaccination and the hesitant group.	0.80
Meppelink *et al.*	2016	NL	Health literacy	Investigate the mechanisms through which health literacy affects information recall and website attitudes.	Website	Limited Capacity Model of Motivated Mediated Message Processing<br>Cognitive Theory of Multimedia Learning	Observational	Adults (821)	1 week (27 February 2015, 5 March 2015)	Campaign effectiveness	• People with higher levels of health literacy need less cognitive capacity to process health information, positively influencing information recall<br>• The relative ease with which health information can be processed positively influ- ences people's evaluation of this information<br>• For people with higher health literacy, it is easier to imagine the content of a health website, which subsequently positively influences attitudes toward the site	• To improve information processing among people with lower health literacy, the cognitive demands of health messages should be reduced, for example by using non-complex texts<br>• The text should be easy to imagine. This could be done, for example, by using images, animations, or concrete language	0.92
Vraga *et al.*	2021	US	Misinformation correction	Test the efficacy of shareable infographics to debunk coronavirus myths, depending on placement and source.	Facebook	5 Cs of correction: is consensus based, includes corroborating evidence, and is consistent, coherent, and credible	Experimental	Participants (1596)	n/a	Message framing	• Exposure to the WHO graphic in any form reduced immediate misperceptions about the science of a false preventive for COVID-19 (that a hot bath can raise body temperature), and this reduction was maintained for at least 1 week for 3 of the 4 correction conditions<br>• The effects were consistent whether the graphic was shared by the WHO itself or by another user.<br>• There is limited evidence that preemptive	• Organizations can debunk misinformation circulating in society by sharing high-quality information on social media emphasizing the facts without waiting to see it shared directly in their feeds, which expands the opportunities for observational correction to occur<br>• Partnerships with platforms may enable automated responses to prominent myths, furthering the reach of expert organizations	0.83

											corrections differ in their effectiveness from reactive corrections		
Vraga *et al.*	2017	US	Misinformation correction	This study tests whether the number (1 vs. 2) and the source (user vs. the CDC) of corrective responses affect successful reduction of misperceptions.	Twitter	n/a	Observational	Students (1,384)	n/a	Campaign effectiveness	• A single correction from a reputable source like the CDC was sufficient to reduce misperceptions<br>• Adding a correction from the CDC after another user has refuted the information again enhances corrective effects<br>• The credibility of the CDC was not harmed when it engaged in this type of corrective action<br>• A single user correction does not produce the same response	Organizations should speak up when they see misinformation on social media. Reputable organizations like the CDC should monitor social media feeds and immediately attempt to rebut misinformation when it arises.	0.77
